# A Distinct Macrophage Population Mediates Metastatic Breast Cancer Cell Extravasation, Establishment and Growth

**DOI:** 10.1371/journal.pone.0006562

**Published:** 2009-08-10

**Authors:** Binzhi Qian, Yan Deng, Jae Hong Im, Ruth J. Muschel, Yiyu Zou, Jiufeng Li, Richard A. Lang, Jeffrey W. Pollard

**Affiliations:** 1 Department of Developmental and Molecular Biology and the Department of Obstetrics/Gynecology and Woman's Health, Center for the Study of Reproductive Biology and Woman's Health, Albert Einstein College of Medicine, Bronx, New York, United States of America; 2 Radiation Oncology & Biology, University of Oxford Churchill Hospital, Headington, United Kingdom; 3 Department of Medicine, Albert Einstein College of Medicine, Bronx, New York, United States of America; 4 Division of Developmental Biology, Department of Ophthalmology, The Children's Hospital Research Foundation, Cincinnati, Ohio, United States of America; Charité-Universitätsmedizin Berlin, Germany

## Abstract

**Background:**

The stromal microenvironment and particularly the macrophage component of primary tumors influence their malignant potential. However, at the metastatic site the role of these cells and their mechanism of actions for establishment and growth of metastases remain largely unknown.

**Methodology/Principal Findings:**

Using animal models of breast cancer metastasis, we show that a population of host macrophages displaying a distinct phenotype is recruited to extravasating pulmonary metastatic cells regardless of species of origin. Ablation of this macrophage population through three independent means (genetic and chemical) showed that these macrophages are required for efficient metastatic seeding and growth. Importantly, even after metastatic growth is established, ablation of this macrophage population inhibited subsequent growth. Furthermore, imaging of intact lungs revealed that macrophages are required for efficient tumor cell extravasation.

**Conclusion/Significance:**

These data indicate a direct enhancement of metastatic growth by macrophages through their effects on tumor cell extravasation, survival and subsequent growth and identifies these cells as a new therapeutic target for treatment of metastatic disease.

## Introduction

Metastatic disease is the major cause of cancer mortality. This fact indicates that metastases are refractory to current treatments such as the traditional modalities of irradiation and chemotherapy as well as the more recent targeted biological therapies. The failure of available treatments suggests that the biological mechanisms that underlie metastatic disease are poorly understood. It is known however, that metastasis is a multi-step process with tumor cells needing to escape from the primary site as well as arriving and prospering at distant sites [1]. In the first steps oncogenic mutations in the tumor cells together with the development of a supportive microenvironment leads to the ability of the tumor cells to invade through the stroma and intravasate into the hematogenous or lymphatic system. These are significant rate limiting steps in the metastatic process [1]. But successful intravasation is only the beginning of the cell's odyssey. Cells destined to produce metastases must survive in the circulatory system, and the distal events of initial seeding and persistent growth in the target organ are also highly inefficient. In fact, senescence or apoptosis of tumor cells entering target organs prevents the lethal spread of the vast majority of intravasated tumor cells [2], [3], [4].

The “seed” and “soil hypothesis by Paget [5] was an early attempt to explain the metastatic process. In its modern guise, the hypothesis proposes that tumor cells have to accumulate sufficient mutations to become metastatic while the target site has to be permissive to allow the appropriately mutated tumor cell to survive and prosper [5], [6]. Recently, many changes in the tumor cell have been identified that allow them to become metastatic and to have a particular trophism to a tissue such as bone or liver [7]. Studies in primary tumors however, have also revealed major roles for the microenvironment in modulating malignancy including significant roles for resident cells such as fibroblasts and adipocytes, as well as haematopoietic cells [8], [9], [10], [11], [12], [13]. Genetic experiments whereby particular classes of cells are ablated in the tumor have revealed major roles for mast cells and macrophages in tumor progression and for macrophages in promoting metastasis [14], [15], [16]. In the latter case macrophages stimulates tumor cell migration, invasion and intravasation in mouse models of breast cancer [8], [9]. These data strongly suggest an active role for an ever-changing microenvironment in the malignant evolution of primary tumors, with macrophages being major players.

Macrophage interactions with metastases have been observed, however their contribution to the metastasis process has been controversial [17], [18]. Recent studies have shown that myeloid cells are involved in primary tumor directed metastatic tumor cell homing to target organs through the creation of preferred sites known as pre-metastatic niches [19], [20]. Studies have also shown that tumor cell secreted factors enhance Lewis lung carcinoma metastasis through their effects on Toll-like receptor 2 signaling in host bone marrow derived cells (BMDCs), including myeloid cells [21]. Nevertheless, as reviewed recently [22], direct roles for macrophages in metastatic growth have not been reported. In this paper using methods that are independent of the influence of the primary tumors, we have tested the hypothesis that macrophages are an important component of the microenvironment that control the survival, migration and growth of metastatic cells. We used different means of macrophage ablation together with an intact lung imaging method and have shown that a population of macrophages with a distinct phenotype is required for metastatic extravasation, survival and growth. Importantly, we show that macrophage depletion even inhibits the growth of established metastatic nodules.

## Results

### CSF-1-regulated macrophages determine metastatic efficiency

**Figure 1:**
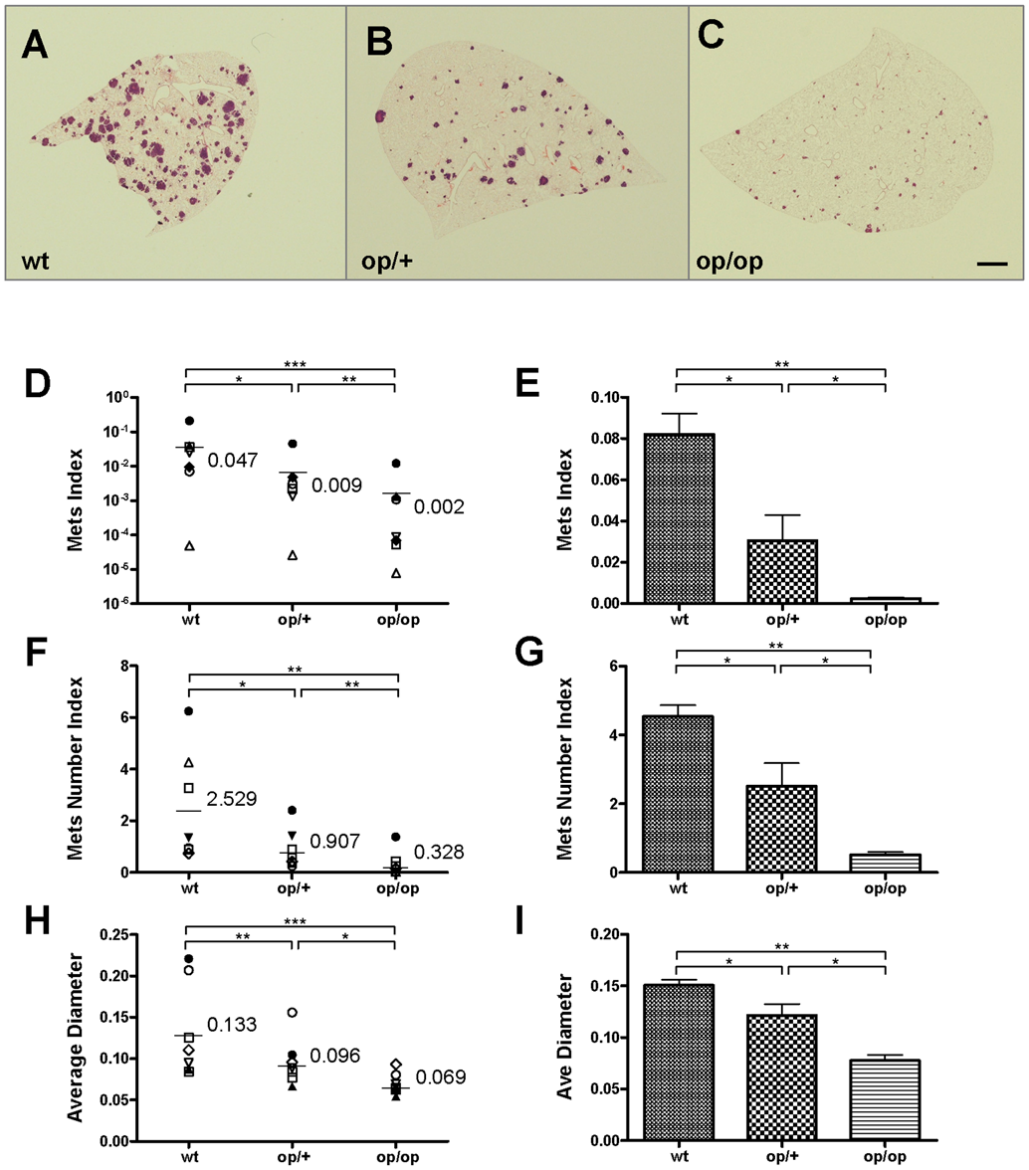
Host CSF-1 determines tumor cell pulmonary seeding and persistent growth. (A–C) Different metastatic potential of primary PyMT induced tumor cells shown in H&E stained transverse section of host lungs of different mouse genotypes. Wild type (wt), heterozygous *Csf1^op^* (op/+) and homozygous *Csf1^op^* (op/op) (D–I) Quantifications of metastasis of primary PyMT tumor cells (D, F and H) and Met-1 cells (E, G and I) using stereological methods. Metastasis index (D and E) is equal to total metastasis volume normalized by total lung volume (note log scale on Y axis in D); Metastasis number index (F and G) is equal to averaged number of metastasis sites per mm^2^ lung area; Average diameter (H and I) is the averaged size of metastasis nodules in millimeter. For primary PyMT cells n = 7, in each graph, data points in mice of different genotypes use the same symbol for each individual tumor. For Met-1 cells data are shown as mean+SEM. n≥4, *p<0.05, **P<0.01 and ***p<0.001.

To test the hypothesis that macrophages regulate tumor cell distal organ seeding and persistent growth, we performed metastasis assays in a well established genetic model of macrophage deficiency. In this model the macrophage deficiency is caused by homozygosity of a null mutation (*Csf1^op^*) in the gene for the major macrophage growth factor, colony stimulating factor-1 (CSF-1) [23]. Initially in these studies, to avoid the potential uniqueness of clonal cell lines, we used primary cultures of tumor cells derived from mammary tumors that had been caused by the mammary specific expression of the Polyoma Middle T oncoprotein from the MMTV promoter [14]. Cultured primary tumor cells prepared as described in the materials and methods were harvested, carefully dissociated into single cell suspensions and injected i.v. into syngeneic (FVB) host mice of different genotypes for *Csf1^op^*. Tumors derived independently from different mice give cells with widely varying metastatic capacity in this assay as assessed in lungs harvested 14 days post-injection and as expected since the tumor stage could not be completely controlled (note the log scale on the Y axis, Figure 1D). However, for every individual tumor cell isolate, a dramatic decline in metastatic capacity was observed according to the allelic frequency for the host *Csf1^op^* mutation (Figure 1A–C). Rigorous statistical measurements using stereological techniques as defined numerically in the Materials and Methods, revealed that the metastatic index for heterozygous mice was significantly lower than +/+ mice and homozygous nulls had a significantly lower metastatic index (total metastasis volume normalized to lung volume) than those containing one or no mutant alleles (Fig. 1D). Both the numbers (shown as metastasis number index, which equals to number of metastasis nodules per mmˆ2 of lung area) (Figure 1F) and the size of metastasis nodules (shown as mm) (Figure 1H) were reduced according to *Csf1^op^* allelic frequency. We have also shown in the past that CSF-1R is expressed in all macrophages and PyMT tumor cells do not express the CSF-1 receptor (CSF-1R) [8], [14] and this was confirmed in the primary isolates of tumor cells used in these experiments by RT-PCR (data not shown). This indicated that the effect of CSF-1 depletion was not due to a direct effect of CSF-1 on the tumor cells themselves but indicates that both metastatic seeding and persistent growth of tumor cells are dependent on macrophages.

To confirm this data with primary tumor cell isolates and to control for the variation observed with primary cell cultures from individual tumors, we repeated the metastasis assay using a highly metastatic cell line, Met-1, derived from PyMT mouse mammary tumor [24]. This cell line is also negative for CSF-1R expression by RT-PCR (data not shown). The data with this cell line confirmed that observed with primary tumor isolates and indicated that the metastatic potential of Met-1 cells was reduced with both seeding and persistent growth being significantly affected according to the host CSF-1 deficiency (Figure 1E, G and I).

The metastatic index is a combination of cell survival and proliferation. Thus to determine the apoptosis and proliferation of the tumor cells in the lungs of mice with different genotypes, we performed TUNEL and Ki67 staining on metastatic lesions 14 days post-injection as indices of apoptosis and proliferation respectively. To avoid variations in non-clonal populations we performed these studies with the Met-1 cell line. These cells showed a significantly increased apoptotic rate according to the host *Csf1^op^* allelic frequency with the homozygous null having the highest rate of apoptosis (Figure S1 A and B). These data were also confirmed with primary tumor isolates indicating that this was not an effect of clonal selection (data not shown). These data suggests that macrophages create an environment in the lung that promotes tumor cell survival.

### Transient macrophage depletion blocks tumor cell metastasis

Mice homozygous for the *Csf1^op^* allele represent a genetic model for macrophage deficiency. However, in this case the deficiency is existent throughout life and it could be argued that the effect on metastasis is secondary to a change in the lung microenvironment caused by macrophage depletion during development. To assess the consequences of an alternative way to deplete macrophages, we used the well-established macrophage-ablation method of treating mice in vivo with liposome encapsulated Clodronate (dichloromethylene diphosphonate) made in-house as described [25]. In parallel experiments performed by our collaborators and us using subcutaneous xenografts showed that there was no uptake of the liposome by PyMT tumor cells [26] and further liposome encapsulated Clodronate did not kill cultured Met-1 cells (data not shown). Consistent with the literature [25] our data showed two injections of Liposomal-Clodronate i.v. was sufficient to deplete macrophages in vivo (Figure S2 and data not shown) and we used liposomal-PBS injection as a control and this had no impact on macrophage numbers (not shown).

**Figure 2:**
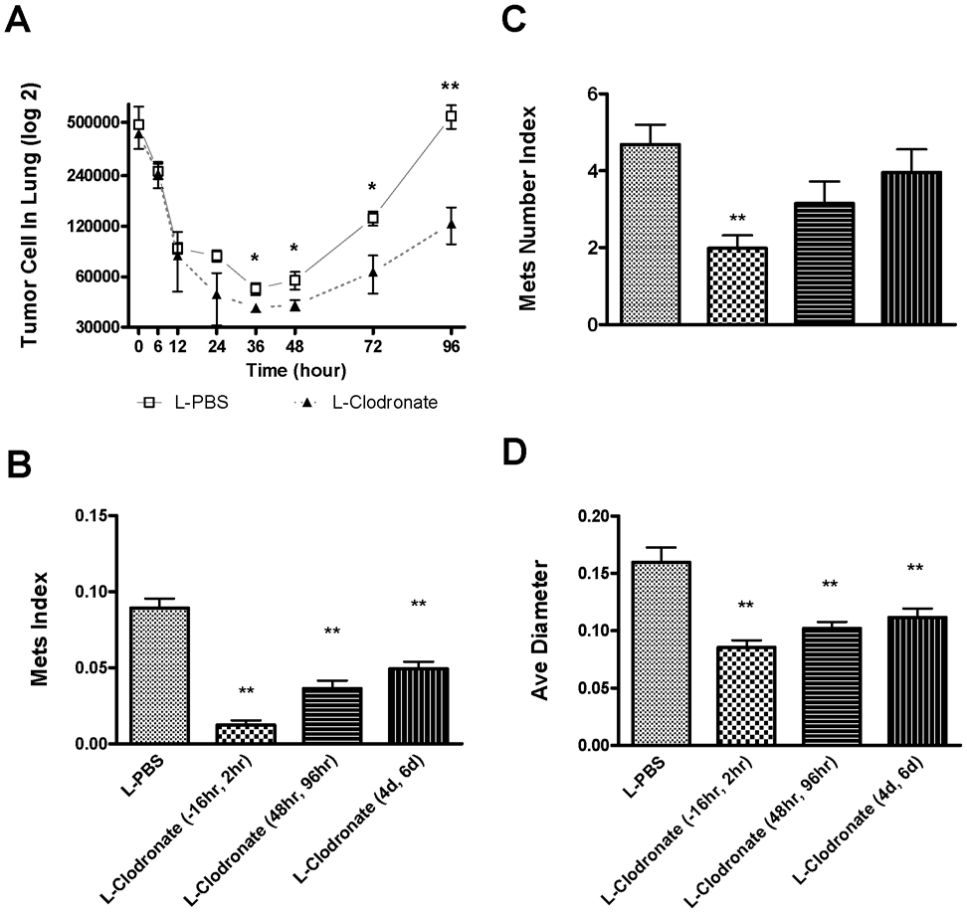
Macrophage depletion blocks tumor cell pulmonary seeding and persistent growth. (A) Absolute number of surviving tumor cells in the lung at different time points indicated after the tail vein injection (n≥3, *p<0.05). Open box, mice treated with liposome-PBS; solid triangle, mice treated with liposome-Clodronate. Data are shown as mean±SEM. (B–D) In vivo macrophage depletion blocks tumor cell pulmonary seeding and persistent growth. In-house prepared liposome-Clodronate was injected i.v. into the tail veins of mice at time indicated according to the time of Met-1 cell injection to deplete macrophage in vivo. Metastasis quantification was the same as in Fig. 1. n≥5, **P<0.01.

Using cumbersome imaging methods some studies have estimated initial tumor cell survival and apoptosis [3], [27]. However, most studies imprecisely measure the final metastatic burden by using end-point assays and only counting the number of metastasis nodules on the lung surface. To understand the initial kinetics of surviving tumor cells once they reach the lung however, we used a precise genomic DNA based quantitative real-time PCR assay for the PyMT transgene (specific for tumor cells but not the host non-transgenic cells) with DNA extracted from whole lung digestions harvested at different time points after i.v. injection of Met-1 cells. In this assay, a standard curve of cell number to Q-PCR cycle threshold was generated so that an exact measure of cell number in the lung could be made. Using this method macrophage depleted mice treated with L-Clodronate were compared to control L-PBS treated mice. In both cases there was no effect on the lodging of tumor cells to the lung with over 85% of the injected tumor cells arriving there immediately after the tail vein injection. In control mice the number dramatically decreases with time until it reaches its nadir at 36 hours after which the cell number starts to increase exponentially. This kinetics clearly marks the seeding phase at around 36 hours post-injection and their initial growth afterwards. In the macrophage depleted mice, tumor cell number over the first 12 hours declined at the same rate as the controls but in contrast to the controls, thereafter it decreases more rapidly with significantly fewer cells remaining in the lung at 36 hours. There was also significantly fewer tumor cells at 48, 72 and 96 hours in the L-Clodronate treated group (Figure 2A). Doubling times of tumor cell growth following this initial 36 hour establishment period was 17.5 hours in L-PBS treated mice and 34.4 hours in L-Clodronate treated mice. Together, these data indicate that macrophages are critical for both seeding and initial growth of metastatic tumor cells consistent with the data using genetic depletion of macrophages.

To verify that the initial kinetics of tumor cell survival is correlated with the final metastatic burden, the same experimental metastasis assay as described in Figure 1 was preformed using Met-1 cells but with L-Clodronate injected at different times post-injection of tumor cells to deplete macrophages at different stages of metastatic development (Figure 2B–D). Compared with the control (L-PBS injection), macrophage depletion over the time of tumor cell i.v. injection (L-Clodronate injection 16 hours before and 2 hours after), greatly reduced the tumor's metastatic potential (Figure 2B), with both seeding and persistent growth inhibited (Figure 2C and D). This is consistent with the lower seeding efficiency and delayed growth of tumor cells in L-Clodronate treated mice measured by Q-PCR method. Importantly depletion of macrophages after tumor cell seeding with L-Clodronate injection given at 48 hr and 96 hr or at 4 and 6 days also caused a reduction in total metastasis burden in both cases (Figure 2B). These treatments significantly limited tumor cell persistent growth (measured by average diameter) (Figure 2D) but not the number of metastatic nodules (Figure 2C). This shows that, as well as seeding of the tumor cells, the growth of established metastatic nodules even once seeding is completed can also be inhibited by macrophage depletion.

### A distinct macrophage population is recruited to lungs bearing metastases

**Figure 3:**
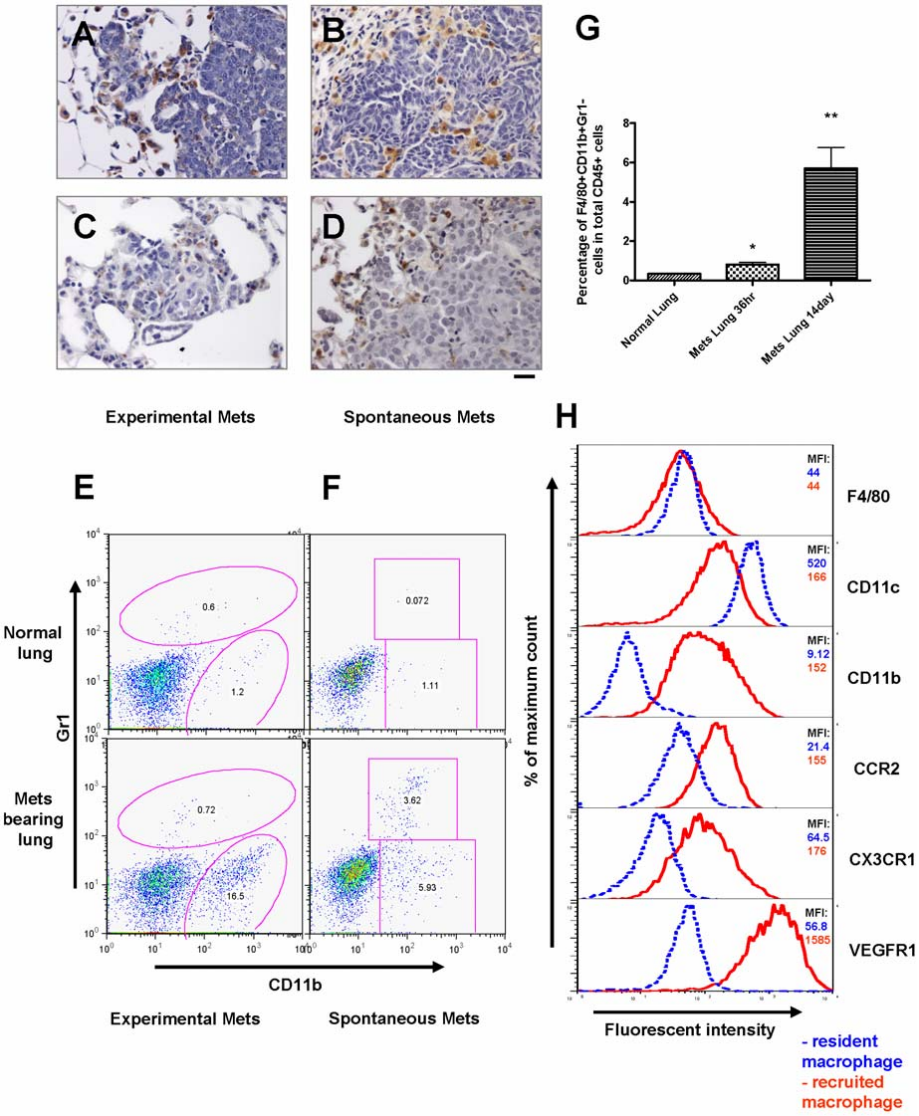
Recruitment of a distinct macrophage population in metastasis bearing lungs. (A–D) Pulmonary metastasis of breast cancer cells are highly infiltrated with macrophages. Representative Mac3 immunohistochemistry staining of transverse sections of lung metastatic lesions from different tumor models: (A) experimental metastasis of primary PyMT tumor cells; (B) spontaneous metastasis derived from a MMTV-PyMT induced mammary tumor; (C) experimental metastasis of Met-1 cells and (D) spontaneous metastasis derived from subcutaneously implanted MDA-231 cells. Bar equals 20 um. (E) Representative flow diagram of CSF-1R-GFP positive cells from normal lung (upper panel) and metastasis bearing lung from experimental metastasis assay of Met-1 cells (lower panel). n = 5 (F) Representative flow diagram of CSF-1R-GFP positive cells from normal lung (upper panel) and lung bearing spontaneous metastasis from MMTV-PyMT induced mouse mammary tumor (lower panel). n = 3 (G) Recruitment of CD11b+Gr1- macrophages (F4/80+) in lungs with experimentally induced metastasis with Met-1 cells. Lungs were harvested at time indicated after tumor cell i.v. injection. Data are shown as mean+SEM. n = 3, *p<0.05 and **P<0.01. (H) Representative flow histograms of normal lung macrophages (F4/80+, blue dashed line) versus recruited macrophage population (F4/80+CD11b+Gr1-, red solid line) from lungs bearing Met-1 cell metastases stained with antibodies of different cell surface makers (indicated at the right side of the histogram). X axis indicates the fluorescent intensity, Y axis indicates the percentage of maximum cell number, MFI (top right panel) denotes representative mean fluorescent intensity (n = 3).

The above data strongly argues for a macrophage population enhancing both seeding and persistent growth of metastatic cells. In mice homozygous for the *Csf1^op^* mutation the lung resident macrophage population is relatively normal in tissue distribution, morphology, number and cell surface markers (Figure S3), consistent with previous reports [28]. This is probably due to the fact that the lung resident macrophages are largely regulated by GM-CSF instead of CSF-1 [29]. In addition, deletion of alveolar macrophage by intra-tracheal injection of L-Clodronate does not affect Met-1 cell metastatic efficiency (data not shown). These data strongly suggest that a distinct macrophage population is recruited from the blood to the metastatic cells in lung. First to establish whether macrophages are recruited to the pulmonary metastases, immunohistochemical staining using anti-Mac3 (a macrophage specific marker [30]) antibody was performed. In the experimentally induced metastasis formed either by primary PyMT tumor cells or Met-1 cells (Figure 3A, C), there was an abundant infiltration of macrophages. This was not a cell-type or route of injection specific phenomena because an intensive macrophage infiltration was also seen in the spontaneous metastases derived from late stage primary tumors of PyMT mice (Figure 3B) and in spontaneous metastasis derived from a subcutaneously implanted human breast cancer cell (MDA-231) in nude mice (Figure 3D). These data show that macrophages are recruited to metastatic lesions regardless of their origin.

Having established that macrophages are recruited to metastatic lesions we next sought to define the characteristics of these macrophages. To this end, we exploited a lineage marked mouse, the MacGreen mouse, in which all macrophages and certain neutrophils are labeled by GFP through expression from the CSF-1R promoter [31] that we have re-derived on an FVB inbred background. We performed FACS analysis of isolated *Csf1r-eGFP* labeled cells and compared these cells from normal lungs with those from lungs carrying experimentally induced metastasis from Met-1 cells. Notably a distinct population of CD11b^+^Gr1^−^ macrophages was consistently recruited by the pulmonary metastasis while this population was essentially absent in normal lungs (Figure 3E). In contrast, other haematopoietic cell populations were not altered significantly by the presence of the Met-1 metastatic lesions (Figure S4). To verify if macrophage recruitment was restricted to experimentally induced metastasis, we compared macrophages from normal lungs with macrophages from lungs with visible spontaneous metastasis in PyMT transgenic mice bearing late stage primary mammary tumors. The same CD11b^+^Gr1^−^ macrophage population was recruited by the spontaneous metastatic lesions (Figure 3F). Thus these cells are recruited to experimentally induced and spontaneous metastases. In combination with another mouse macrophage marker, F4/80 [32], we verified that these cells are macrophages and that a significant recruitment to the lungs appeared as early as 36 hours after introduction of the tumor cells into the circulation (Figure 3G). Detailed immunophenotyping of this CSF-1R+population shows that compared to normal lung resident macrophages (which are CD11c^+^), this distinct CD11b^+^ Gr1^−^ macrophage population has similar F4/80 expression, dim CD11c^+^ expression, and strong CX3CR1, CCR2 and VEGFR1 expression (Figure 3H). These cells do not have detectable surface Tie2 or CXCR4 expression (data not shown), which makes them distinct from previous identified pro-angiogenic macrophages 33, 34. Both lung resident macrophages and metastasis recruited macrophages also have similar Mac3 expression verifying the IHC staining (Figure S5).

### Deletion of CD11b+ macrophages in vivo inhibits metastasis

**Figure 4:**
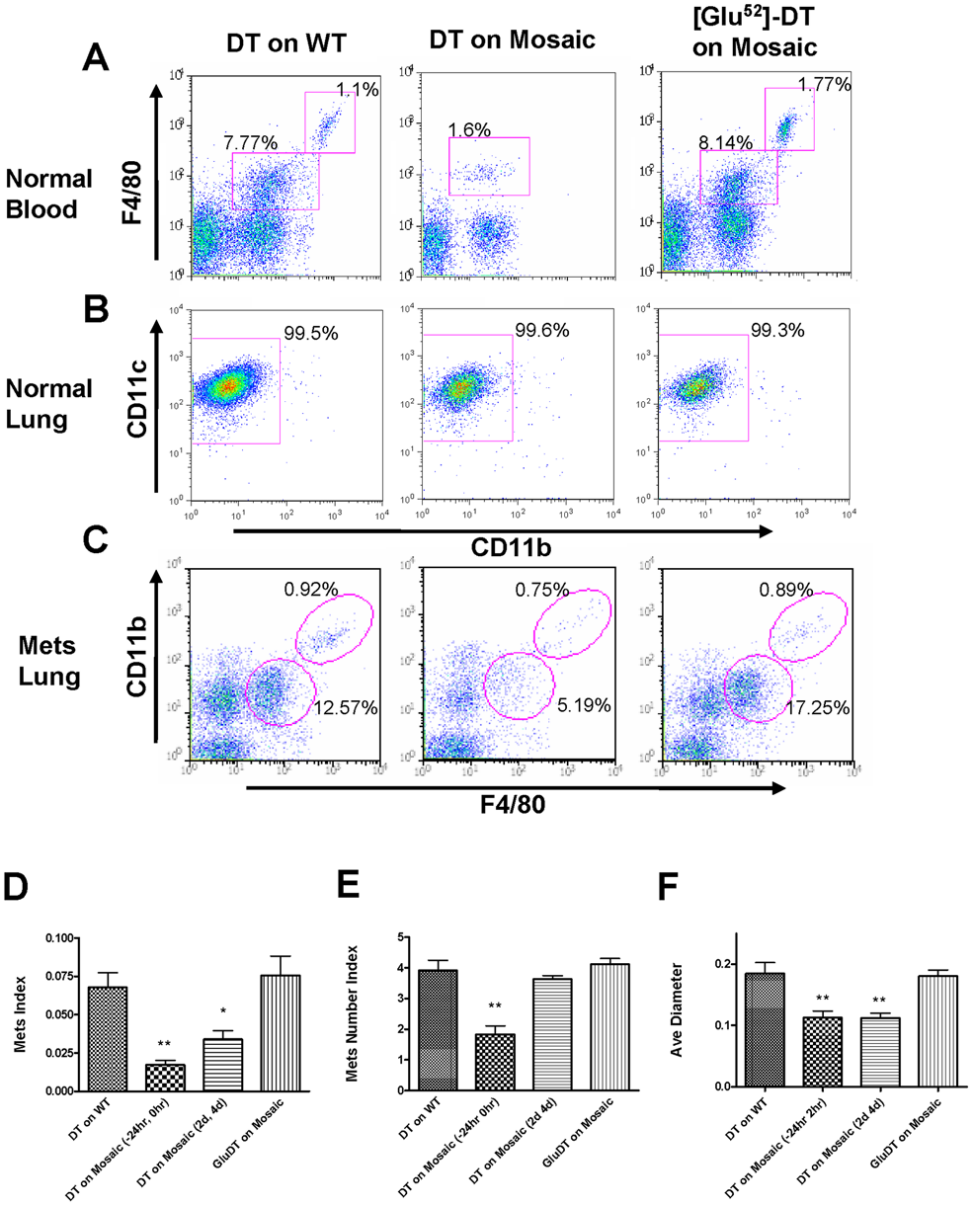
Ablation of CD11b+macrophages blocks tumor cell pulmonary seeding and persistent growth. (A) Representative flow diagram of blood CD45+cells in mice treated with diphtheria toxin (DT) or Glu^52^ mutated DT. X axis, CD11b expression; Y axis, F4/80 expression. (B) Representative flow diagram of lung F4/80+cells in mice treated with DT or Glu^52^-DT. X axis, CD11b expression; Y axis, CD11c expression. (C) Representative flow diagram of lungs CD45+cells of mice with experimentally introduced Met-1 cell metastases before DT or Glu^52^-DT treatment. X axis, F4/80 expression; Y axis, CD11b expression. (D–F) In vivo depletion of CD11b+macrophage blocks tumor cell pulmonary seeding and persistent growth. DT was given i.p. at the times indicated according to Met-1 cell injection and metastasis quantifications were the same as in Fig. 1. DT treatment on mice with wild type bone marrow transplant and Glu^52^-DT treatment on mosaic mice were used as controls. Data are shown as mean+SEM. n≥5, *p<0.05 and **P<0.01.

To test whether the recruited CD11b+ macrophage population has an important role in promoting metastasis, we took advantage of a transgenic mouse expressing the human diphtheria toxin (DT) receptor (DTR, also known as heparin-binding EGF; hb-EGF) under the control of the a truncated mouse CD11b promoter (CD11b-DTR), in which CD11b+F4/80+macrophages but not neutrophils (that also express CD11b) can be conditionally ablated upon DT injection [35]. To avoid problems in interpretation of potential leaky expression of the transgene in non-haematopoietic cells, we generated mosaic mice by bone marrow transplant using CD11b-DTR mice as the bone marrow donors. Wild type into wild type bone marrow transplants were used as transplantation controls while a mutated inactive form of the DT (Glu^52^-DT) was used as toxin control [36]. As expected, two DT injections given over 48 hours greatly depleted the CD11b+macrophages in the peripheral blood in the mice that received the transplantation of bone marrow from CD11b-DTR mice, but had no effect on these populations in wild type bone marrow transplant controls. Similarly Glu^52^-DT treatment has no effect on the transgene mosaic animals showing the depletion is toxin dependent (Figure 4A). Other haematopoietic cell populations were not significantly affected by DT treatment consistent with the macrophage restricted expression of the truncated promoter (Figure S6). In the lung, resident CD11c+macrophages were also not affected by the DT injections consistent with their lack of CD11b expression (Figure 4B).

Since CD11b+macrophages are recruited to the metastasis-bearing lungs, we analyzed whether these cells are depleted following DT injection. DT was injected at a time when significant metastases were induced by Met-1 cells injection and when CD11b cells are already recruited. As expected, CD11b^+^ macrophages (F4/80^+^) were specifically depleted in the peripheral blood and in the metastasis-bearing lungs (Figure 4C and Figure S7C). Another F4/80^high^CD11b^high^ population, which has been reported to be involved in B cell immune regulation [37], was unaffected by this treatment however, suggesting that the transgene was not being actively transcribed or translated in this population despite the cells having CD11b expressed from the endogenous gene (Figure 4C and Figure S7C). A change of CD11b+Gr1+bone marrow derived suppressor cells probably pre-granulocytes [38] were believed to be responsible for the reduced metastasis of Lewis lung carcinoma cells in TLR2−/− mice [21]. However in our metastasis model, these cells are F4/80- and their recruitment is not significantly different in metastasis bearing lungs compared with normal lungs (data not shown). Furthermore, they are not affected by DT treatment in metastasis bearing lungs (Figure S7A and B)

Having established the potency of this diptheria transgene method in depleting CD11b^+^ cells, we used it to deplete CD11b^+^ macrophages co-incident with the tumor cell injection (24 hours before and 2 hour after injection). Compared with DT treatment on wild type bone marrow transplant control, this treatment greatly reduced the tumor cell metastatic potential, with both seeding and persistent growth impaired (Figure 4D, 2^nd^ histogram). When the CD11b^+^ macrophage population was depleted after tumor cell seeding had occurred (treated 2 and 4 day after tumor cell i.v. injection) only the persistent growth of metastatic tumor cells was reduced (Figure 4D, 3^rd^ histogram), consistent with the effects of macrophage depletion using L-Clodronate described above. Treatment of bone marrow transplanted transgene-bearing mice with the mutated toxin had no effect on tumor metastastic potential (Figure 4D, 4^th^ histogram). These data confirm the requirement of macrophages for metastatic seeding and persistent growth and further identify the recruited CD11b+ population as the effector cells.

### Host CD11b+Gr1- macrophages are critical for human breast cancer cell experimental metastasis

**Figure 5:**
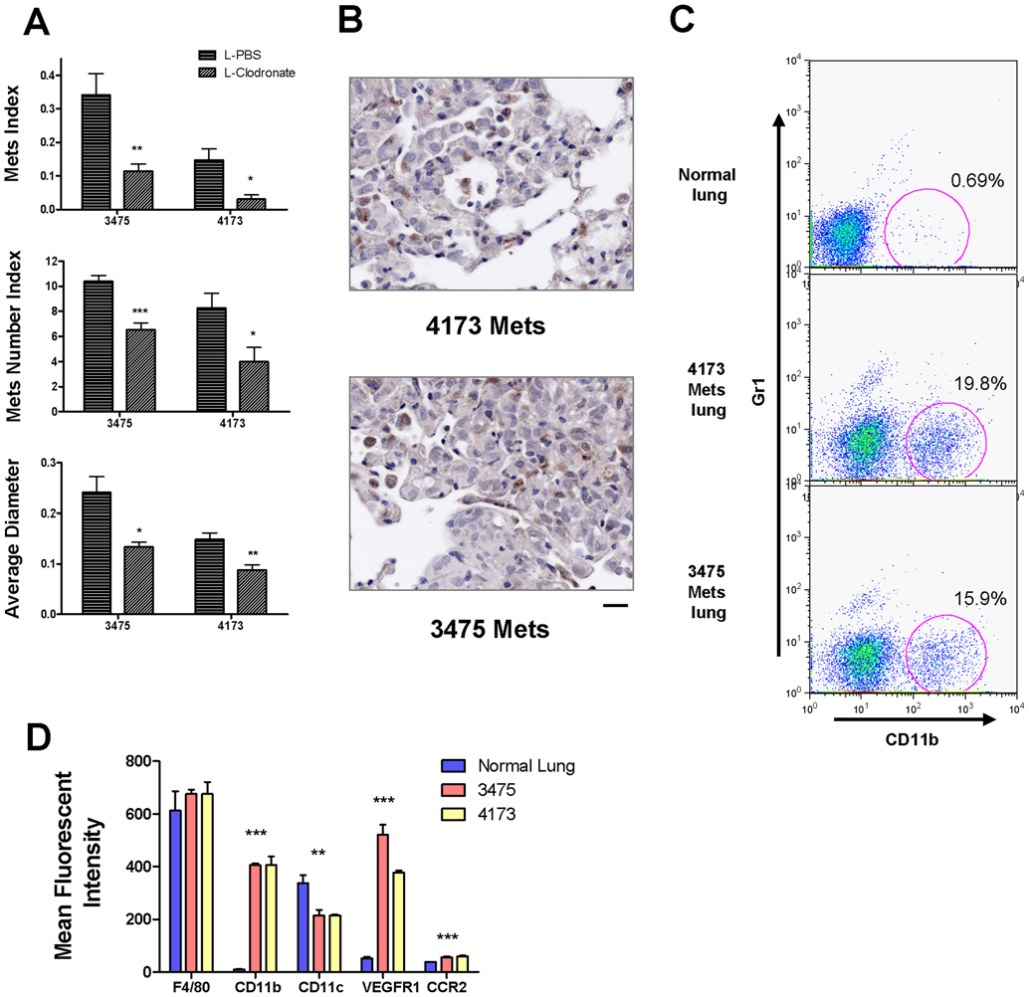
Host CD11b+Gr1- macrophages promote human breast cancer cell experimental metastasis. (A) In vivo macrophage depletion by liposome encapsulated Clodronate blocks tumor cell pulmonary seeding and persistent growth of MDA-231 derived human breast cancer cell lines, 3475 and 4173. Metastasis quantifications were the same as in Fig. 1. Data are shown as mean+SEM. n = 5, *p<0.05, **P<0.01 and ***p<0.001. (B) Pulmonary metastases of human breast cancer cells in nude mice are highly infiltrated with macrophages with anti-Mac3 antibody staining as described in methods. (C) Representative flow diagrams of CD11b+Gr1- cells recruitment by pulmonary metastases of 4173 and 3475. Lung F4/80+cells were separated by surface expression of CD11b (X axis) and Gr1 (Y axis), n = 3 (D) A graph comparing flow cytometric data of mean fluorescent intensity of different cell surface markers (F4/80, CD11b, CD11c, VEGFR1 and CCR2) expressed by normal lung macrophages (left histogram) and macrophages recruited by pulmonary metastases of 3475 (middle histogram) and 4173 (right histogram). Data are shown as mean+SEM. n = 3, *p<0.05, **P<0.01 and ***p<0.001.

To test the hypothesis that macrophage dependency is a general phenomena for breast cancer pulmonary metastasis, experimental metastasis assays were performed in nude mice using the metastatic variants of MDA-231 human breast cancer cell lines, 3475 and 4173 (kind gifts from Dr. Joan Massague (MSKCC, New York) [39]. In mice whose macrophages were depleted with L-Clodronate, the metastatic potential of both cell lines was significantly reduced, with both seeding and persistent growth impaired (Figure 5A). Established metastatic nodules were also highly infiltrated with macrophages (Figure 5B). Comparing F4/80+ macrophages from metastasis-bearing lung with those from healthy lungs, it was observed that a significant population of CD11b+Gr1- macrophage population was recruited by the metastases derived from both cell lines (Figure 5C). This macrophage population has similar F4/80 expression with macrophages from healthy lungs, but has a distinct phenotype of CD11c^dim^ CD11b+VEGFR1^high^ and CCR2^high^ (Figure 5D). In addition, there is another but uncharacterized F4/80+ population that is Gr1+ but CD11b-. The identity and function of these cells is unknown but they are unique to these human cell lines. Nevertheless, in metastasis derived from human breast cancer cells, we can conclude that the same population of macrophages as found in mouse metastases is required for efficient tumor cell seeding and persistent growth.

### Tumor cell -macrophage interaction is required for tumor cell extravasation

**Figure 6:**
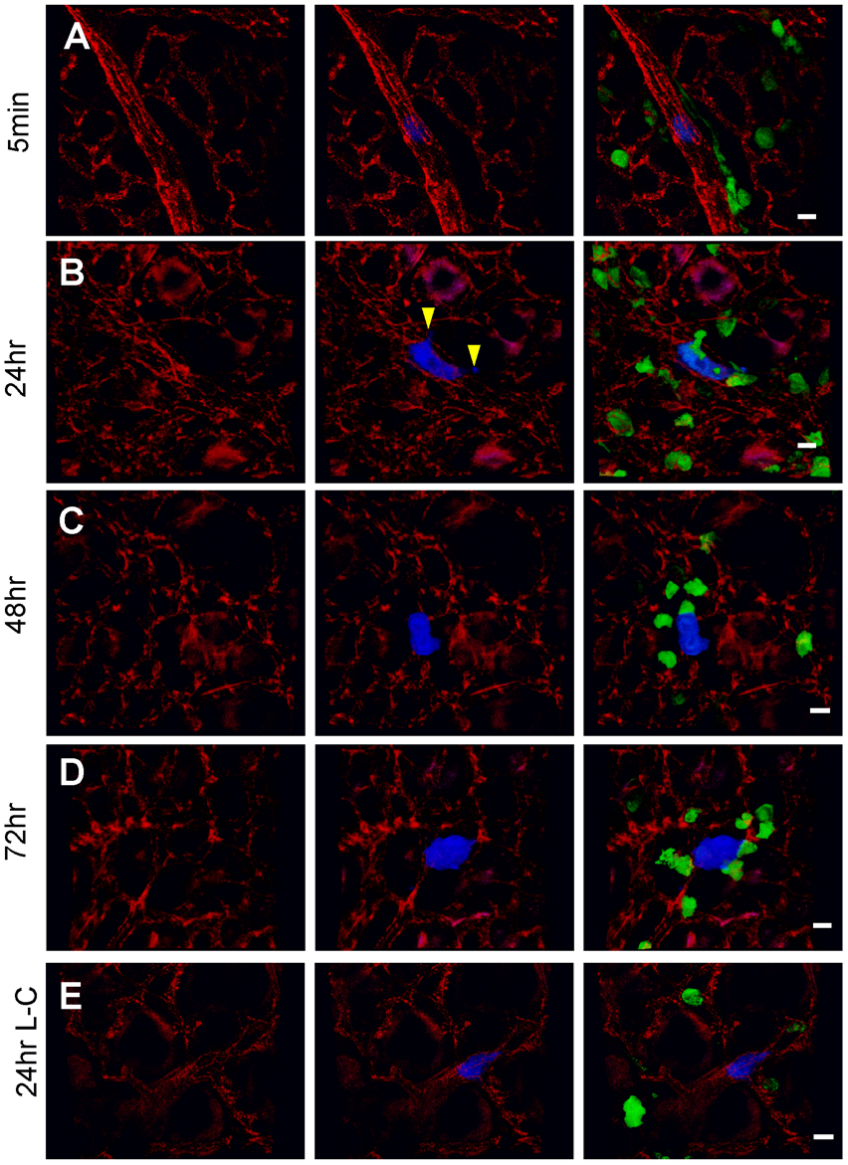
Tumor-macrophage interaction in the lung using ex vivo imaging. Representative snapshots of 3D reconstructed confocal images of tumor cell (CFP, shown in blue) and macrophage (GFP, shown in green) at different times indicated after tumor cell tail vein injection: 5 minutes (A), 24 hours (B), 48 hours (C), 72 hours (D) and 24 hours in L-Clodronate treated mouse (E). Blood vessel were stained with Alexa Fluor® 647 conjugated anti-mouse CD31 antibody (shown in red). Bar equals 20 um. Arrow heads in B indicate the extravasated part of the tumor cell.

Despite the considerable efforts that have been expended on studying the interaction between metastasizing tumor cells and blood vessel in target organs, no uniform conclusions have been reached [40], [41]. Thus to explore the role of the macrophages in metastatic seeding and extravasation mechanistically, we used an intact ex-vivo lung imaging system that preserves the physiological lung structure [42]. In this system, *Csf1r-EGFP* transgenic mice were injected i.v. with CFP-expressing Met-1 cells and harvested at different times after injection. Dye conjugated anti-mouse CD31 antibody was injected i.v. to label blood vessels. Fluorescent images of the resultant lungs were taken using a Leica® confocal microscope and 3D reconstructions that allowed precise measurements of cell extravasation, macrophage number and area of direct interaction with tumor cells were performed using Volocity™ software. Representative images of at least three separate experiments are shown in Figure 6A–E and 3D reconstructed images are shown in supplemental movies.

**Figure 7:**
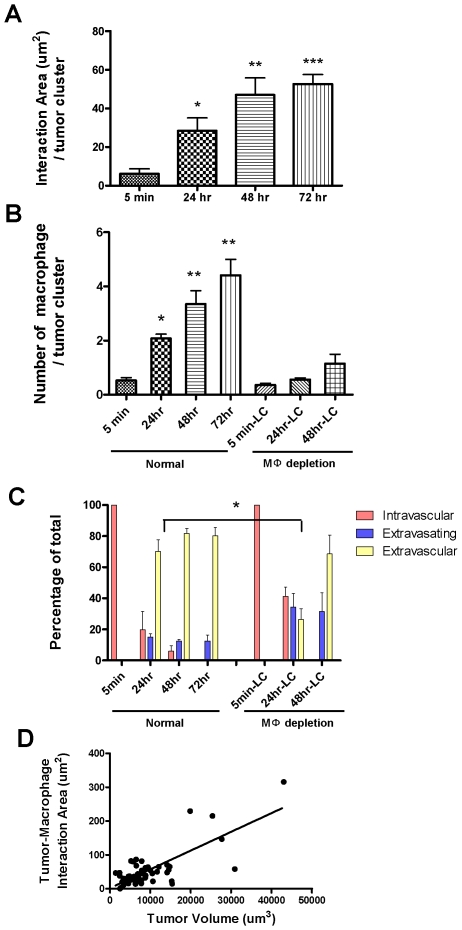
Tumor-macrophage interactions promotes tumor cell extravasation and correlates with initial tumor growth. (A) Average area of direct interaction between tumor cells and macrophages per tumor cluster measured by confocal microscopy at different time points after iv injection. Statistically different from 5 min time point *p<0.05, **P<0.01 and ***p<0.001 (B) Left four histograms: Average number of macrophages that directly interact with each tumor cell cluster at different time points after iv injection. Statistically different from the 5 min control *p<0.05, **P<0.01. Right three histograms: Interactions following macrophage depletion using Liposome-Clodronate administered 16 hrs before and 2 hours after iv injection as described in the materials and methods. All time points are significantly lower than the macrophage replete mice. (C) Tumor cell extravasation status at different time points after iv injection showing the percentage of totally intravascular (red), extravasating (blue, inside vessels and outside) and extravascular (yellow, completely outside vessels) in normal or macrophage depleted mice using Liposome-Clodronate as described above. Note that data is represented as a percentage of total cells although the number of viable tumor cells is greatly reduced after macrophage depletion (Fig. 2A). The delay in extravasation at 24 hrs following macrophage depletion is statistically significant with p<0.05. (D) Correlation between tumor cluster volume and tumor-macrophage interaction area at 72 hours after tumor cell tail vein injection. p<0.0001, R^2^ = 0.58 (A–C are based upon 3D images of 10–20 clusters per animal, 3–6 mice per time point. Data are shown as mean+SEM. D., 51 tumor clusters from 3 mice).

Five minutes after tumor cell injection (Figure 6A, Movie S1), numerous tumor cells have reached the lung vasculature consistent with the Q-PCR data (Figure 2A). Most tumor cells were not interacting with lung resident macrophages, although a few are attached in the vessel to GFP-labeled cells with morphology consistent with monocytes (Figure 7A left most histogram). At five minutes, all tumor cells were retained in the vessel (Figure 7C left most histogram). Some blood vessels were much larger than a single cell diameter, suggesting active attachment of the tumor cells rather than physical restraint although they may have been surrounded by platelets that would not have been visualized in these experiments. At 24 hours (Figure 6B and Movie S2), the number of tumor cells remaining in the lung dramatically decreased, consistent with the Q-PCR data. Importantly at this time tumor cell and macrophage interactions dramatically increased, measured by both the number of macrophages interacting with each tumor cluster and the contact area between these two cell types (Figure 7A and B). Interestingly, ∼75% of the surviving tumor cells are outside the vessel at this time (Figure 7C). Tumor cells under-going extravasation (crossing the vessel) were observed, with most of the extravasated part (Figure 6B, arrow head) directly interacting with macrophages (Figure 6B and Movie S2). At 48 and 72 hours, the majority of the tumor cells were outside the vessel with an increasing number of interacting macrophages (Fig 6C, D, Movie S3 and 4). At 72 hours, no cells were observed to be completely inside the vessel (Figure 7C). Some tumor clusters are larger than a single cell in dimension suggesting cell proliferation, and these clusters have extensive macrophage interactions (Figure 6D, 7D and Movie S4). Starting from 24 hours, tumor volume is positively correlated with macrophage interaction area (Figure 7D and data not shown).

To further analyze the role of macrophages in these early processes, similar experiments were performed but in this case following macrophage depletion using L-Clodronate. Consistent with the data in Figure 2 there were many fewer tumor cells observable in the lung following macrophage depletion. Furthermore those that remained had virtually no interaction with macrophages compared to that seen in control mice (Figure 7B). Importantly the extravasation of tumor cells was delayed following macrophage depletion such that at 24 hrs only ∼25% were extravascular compared to ∼75% in the control mice and with twice the number still entirely retained in the vessels (Figure 6E, 7C and Movie S5). By 48 hrs those few tumor cells that have survived have mostly extravasated although significantly more are still in process (Figure 7C). In contrast, tumor cell behavior in L-PBS treated mice did not differ from untreated mice (data not shown). These data therefore identify a unique mechanism for macrophages in promoting tumor cell extravasation into the metastatic site. In addition, these data combined with that in Figure 2, show that tumor cells begin to proliferate once extravasated and this proliferation is promoted by the association of macrophages within the establishing metastatic nodules. Together these data indicate that macrophages promote the earliest steps in metastatic seeding in that they significantly enhance the rate of tumor cell extravasation and survival.

## Discussion

There is a growing body of evidence that indicates that macrophages in the primary tumor promote tumor progression to the metastatic phenotype [43]. This evidence comes from two sources: 1) clinical correlative data that shows in over 80% of the cases that a strong macrophage infiltrate is correlated with poor prognosis [44]; 2) mouse models where genetic ablation of macrophages results in an inhibition of tumor progression and a reduced rate of metastasis [14]. In part, this tumor promotion by macrophages is due to their role in regulating angiogenesis [45] and to their ability to enhance tumor cell motility, invasion and intravasation (reviewed in [11]). These macrophage activities therefore increase the potential for metastatic spread from the primary tumor not only by stimulating tumor invasiveness but also increasing the number of target vessels through which the tumor cells escape. These tumor-associated macrophages have been described as trophic macrophages [46] or M2 [47] since they have a phenotype that suggests developmental, tissue remodeling as well as immune-regulatory functions that suppress cytotoxic immune responses [48], [49], [50].

Macrophages have also been found associated with tumors at metastatic sites [26]. Earlier studies attempted to determine macrophage functions in distal metastasis events by their co-injection with tumor cells. Although the injection of tumor cells mimics the large number of tumor cells that are shed from late stage tumors [51] mature macrophages are not observed in the circulation. Thus this system remains artificial and contradictory results have been observed [17], [52]. Recent studies however have indicated that bone marrow derived cells in addition to their roles in the primary tumor also promote metastasis through their effects at secondary sites. In particular, it has been shown that primary tumors influence the selection of metastatic sites through the secretion of factors that recruit bone marrow cells to these sites to create the so-called pre-metastatic niche in which circulating tumor cells settle and prosper [53], [54]. The bone marrow cells that populate these niches have not been fully characterized but are of myeloid origin [55]. Despite these important studies, there remains no direct evidence for macrophages influencing the events subsequent to homing of metastatic cells [22]. To test whether macrophages do have a role in these subsequent events, we used an experimental metastasis model to circumvent the influence of primary tumors. Using this system we show that macrophage play a significant role in the extravasation of metastatic cells as well as in their establishment and growth in the lung.

This conclusion that macrophages have a major impact on metastatic cell seeding and persistent growth was based upon studies using three different and independent methods of macrophage ablation. The first used mice carrying a null mutation (*Csf1^op^*) in the major macrophage growth factor, CSF-1, to deplete the macrophages [56] and these studies showed a profound inhibition of metastatic cell seeding and persistent growth. Interestingly, there was an effect on the metastatic index (a sum of metastasis number and size) according to the null allele frequency. This is unusual since previous studies showed that heterozygous mice have normal serum concentrations of CSF-1 and normal populations of macrophages in most tissues tested [57]. Indeed in the lung we showed that+/*Csf1^op^* mice also have normal resident macrophage numbers (Figure S3). However, a radioimmuno-assay of lung tissue CSF-1 revealed a reduced CSF-1 expression in the heterozygote (data not shown). This indicates the strong dependence of host CSF-1 level for efficient tumor cell seeding and growth in lung. It will be interesting to determine if a heterozygote effect can be found in human populations with breast cancer. It should be noted that increased levels of circulating CSF-1 in human patients with breast, ovarian and endometrial cancer is correlated with poor prognosis [58], [59], [60].

The second method of macrophage depletion used the classical method of liposome-encapsulated Clodronate that causes macrophage death after its selective phagocytosis by mature macrophages [25] but not by tumor or other cells [26]. When the macrophages were depleted by this method spanning the period of metastasis assay, both tumor cell seeding and persistent growth were inhibited. Importantly macrophage depletion using this method after metastatic seeding resulted in the reduction of metastatic growth.

The third method was based upon the observation (see results and below) that the recruited macrophage population was different from the lung resident CD11c+ macrophages in their expression of CD11b. This enabled the use of a suicide gene approach whereby the CD11b+positive macrophages were ablated by dint of their expression of the diptheria toxin receptor from the CD11b promoter [35]. Using this method we showed that this CD11b+population of macrophages was required for both seeding and persistent growth. Importantly the resident population of macrophages were unaffected by this treatment showing that it is the newly recruited population that is responsible for the effects. Furthermore similar to the data obtained following L-Clodronate ablation, depletion of these macrophages using the diptheria toxin approach after the metastasis had been established also resulted in inhibition of growth. Thus we can conclude from these three approaches that the CD11b+population of mature macrophages that is recruited to the lung in response to the arrival of metastatic cells promote their subsequent establishment and growth. This ability to inhibit the growth of metastases once established also suggests that macrophage depletion will have significant therapeutic potential.

Our data showed the recruited macrophage population is characterized by a cell surface marker signature of F4/80+CSF-1R+CD11b+Gr1-CX3CR1^high^CCR2^high^ and VEGFR1^high^ and their recruitment to pulmonary metastases is independent of the metastatic cell type or the species of origin (mouse and human). It should also be noted that although this experimental model of metastasis is somewhat artificial in that a bolus of cells arrives to the lung within 5 minutes, a population of macrophages with a similar phenotype is also recruited to spontaneous metastases derived either from primary autochthonous mammary tumors or from xenotransplants of human breast cancer cells in immunocompromized mice. These macrophages display characteristic markers such as F4/80, Mac3, CSF-1R and are phagocytic because they up-take liposomes (see below) while being negative for granulocyte marker, Gr1. While none of these markers are unique to macrophages in themselves, the combination and their high level of expression together with the cells tissue location indicates that these cells are definitive macrophages [46]. They probably differentiate from monocytes precursors that are restricted to blood and seed most macrophages but it cannot be ruled out that they cross-differentiate from other resident macrophages as has been described in some immune responses [61]. This metastasis-associated CD11b+macrophage population is different from the classically defined inflammatory Gr1+CCR2+CX3CR1^low^ macrophages and Gr1-CCR2-CX3CR1^high^ tissue macrophages [62] and is also distinct from other recently identified macrophage populations in the primary tumor microenvironment such as myeloid suppressor and pro-angiogenic macrophages [11], [63], 64. It is also different from the populations found in the primary tumors of PyMT mice in the expression of CXCR4 and Tie2 (our unpublished data). They are however, similar to a recently identified anti-inflammatory macrophage important in facilitating myogenesis in terms of Gr1 expression [65]. This distinct phenotypes give further support to the notion of the tumor microenvironment educating the recruited macrophages to give functions that are advantageous to the growing tumor cells [66].

Our data shows that macrophages not only affect metastatic growth but also seeding. This ability of tumor cells to establish themselves at the metastatic site is considered one of the major rate limiting steps in metastasis [1]. But there is considerable controversy about how tumor cells interact with blood vessels in their metastatic target organ and the subsequent steps in establishment [40]. Because of the unique vasculature in the lung, conventional method of vessel labeling using dextran does not work well since this molecule leaks out easily. Furthermore, because lung is fragile, the vessel structure is often damaged during fixation and sectioning. Thus to examine the early steps we used an intact lung imaging system [42] with methods that visualizes macrophages, blood vessels and tumor cells simultaneously followed by detailed quantitative analysis of extravasation events together with a QPCR method that accurately measures tumor cell number. For these methods single cell suspensions were carefully prepared to avoid emboli formation and these were not observed in the lung vasculature after tumor cell injection. Indeed as the individual tumor cells arrive in the lung they begin a process of attachment and invasion. While this process is inefficient the presence of the tumor cells stimulates the recruitment of macrophages that form intimate contacts with them as soon as they extrude through the vessel walls. Importantly, the rate of extravasation of the tumor cells was significantly reduced after macrophage depletion with a co-incident reduction in tumor cell viability. Once extravasation is completed tumor cell proliferation begins and there is a positive correlation of tumor cell growth with macrophage association at these early stages. Consistent with this role of macrophages in promoting growth, macrophage depletion at this time resulted in a two-fold increase in the population doubling time. Thus this novel imaging technique together with rigorous quantification shows that extravasation can be a rate limiting step in the metastatic process and also identifies this population of macrophages as a component of the microenvironment that plays a critical role in this step as well as in the subsequent growth of the surviving tumor cells. Further this imaging of early stage events in metastasis is consistent with our end point stereological measurements that show macrophages positively influence both tumor cell seeding and persistent growth.

**Figure 8:**
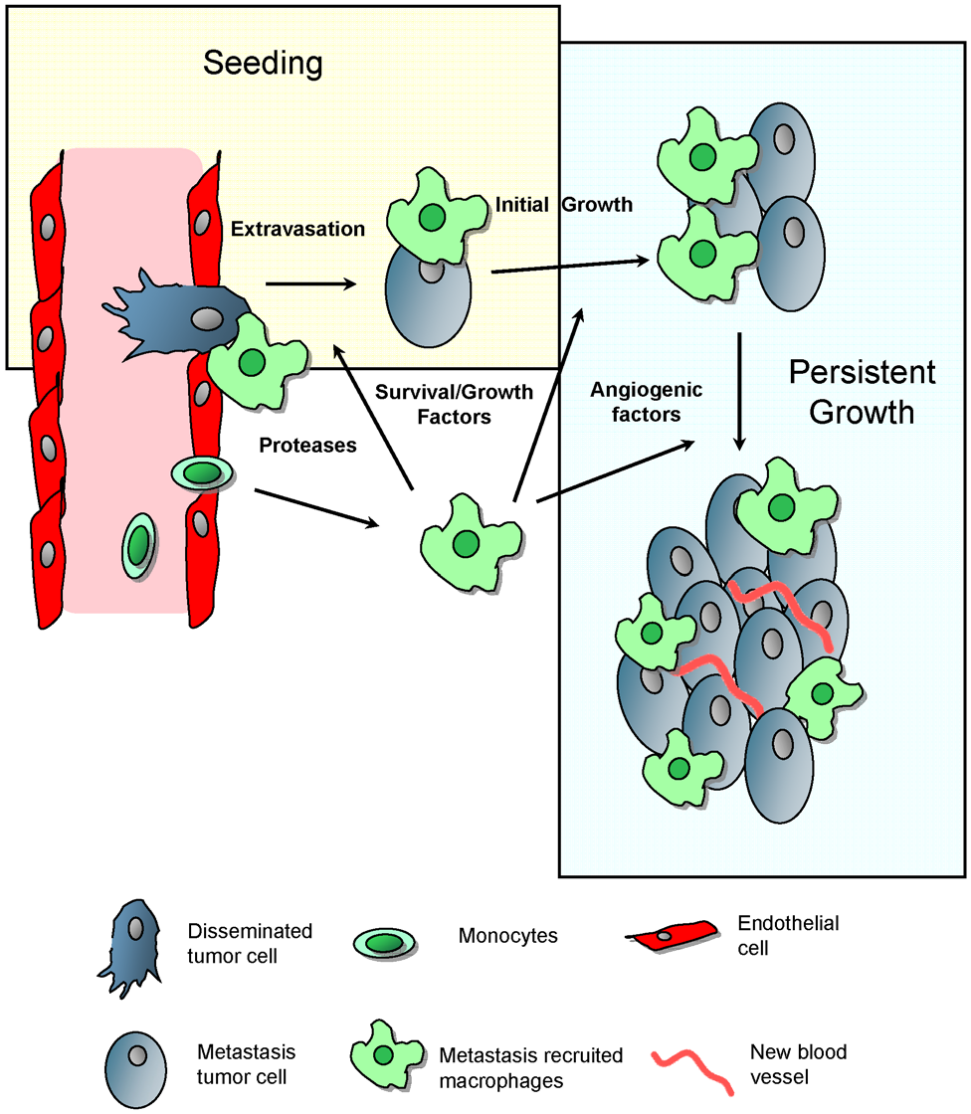
Model for macrophage promotion of metastasis at distant sites. Based on the data in current study, we suggest a model for the macrophage enhancement of metastasis at the distal target organ. Following arrest of the tumor cells in capillaries of metastasis target organ, monocytes are quickly recruited and differentiate in situ into metastasis associated macrophage phenotype whereafter they promote the different steps of metastatic seeding, initial and persistent growth as indicated in the figure and described in the discussion. Figure modified from Joyce and Pollard, 2009.

Metastasis remains an intractable problem clinically and is therefore the major cause of death in cancer patients. Based on the data in current study, we suggest a model for the macrophage enhancement of metastasis at the distal target organ (Figure 8). Following arrest of the tumor cells in capillaries of metastasis target organ, monocytes were quickly recruited and differentiated in situ into metastasis associated macrophage phenotype with a distinctly defined cell surface marker phenotype. This recruitment is at least in part under the influence of locally synthesized CSF-1, a well-documented growth and differentiation factor for macrophages. In addition as these macrophages express receptors for CCL-2 and VEGF (CCR2 and VEGFR1 respectively), both cytokines that are chemotactic for macrophages, it is likely that such signaling molecules will play a role in this recruitment process. These CD11b+macrophages recognizes extravasating tumor cells and our imaging shows that they interact with them directly and help them invade into the lung parenchyma. This is presumably through the secretion of proteases, growth, and motility and survival factors. In the absence of these newly differentiated macrophages, this process of tumor cell extravasation is very inefficient and the tumor cells rapidly die by apoptosis and thus the seeding efficiency is very low. Once extravascular the tumor cells continue to send signals to recruit and also possibly influence the differentiation of the macrophages into trophic ones [46] that further enhance tumor cell viability and growth. When the tumors attain a certain size these macrophages are also likely to provide angiogenic factors as they have been documented to do in the primary tumor [45] that then help in the vascularization needed for continuous metastatic growth. In this scenario several macrophage signaling pathways and functions are likely to be engaged at the different steps of tumor cell seeding, initial and persistent growth. These are continuous process and overlapping processes as ablation of macrophages after the metastatic lesions are established retards their growth significantly. These data suggests that macrophages themselves or their unique signaling pathways represent new therapeutic targets that may be efficacious in reducing cancer mortality.

## Materials and Methods

### Animals

#### Ethics statement

All procedures involving mice were conducted in accordance with National Institutes of Health regulations concerning the use and care of experimental animals. The study of mice was approved by the Albert Einstein College of Medicine Animal Use Committee.

All transgenic mice were kept on the FVB background. Transgenic mice expressing the PyMT oncogene under the control of MMTV LTR were provided by Dr. W.J. Muller (McMaster University, Ontario, Canada) and bred in house. Detailed descriptions of the origin, care, and identification of *Csf1^op^/Csf1^op^* (designated op/op in figures) mice and their heterozygote controls have been given previously [57]. *Csf1^o^/^op^* mice were backcrossed into FVB background in house for 10 generations (courtesy of E. R. Stanley, Albert Einstein College of Medicine). Then male *+/Csf1^op^* mice were randomly bred with +/*Csf1^op^* females to obtain female mice of *Csf1^op/op^*, +/*Csf1^op^* or +/+background. *Tg*(*Csf1r-EGFP*)*Hume* mice have been previously reported to have the whole mononuclear phagocyte system labeled [31] and were remade on the FVB background FVB.(*tg(Csf1r-EGFP)1Jwp)* with similar results. Transgenic mouse expressing human hbEGF driven by CD11b promoter (*tg(CD11b-DTR)Llan*) is a previously reported model for effective in vivo transient CD11b-expressing macrophage depletion. [35], [67] Homozygous animals were bred in house and were used as bone marrow donors to generate chimeras with lethally irradiated 3 week-old FVB females (Charles River, MA). Control chimeras were generated using wild type bone marrow donor. Diptheria toxin (DT) (Sigma-Aldrich, MO) was given i.p. at a dose of 50 ng per gram of body weight in PBS, and Glu52-DT was give at the same dose.

### Cell Culture

All cells were cultured in Dulbecco's modified Eagle's medium (DMEM) supplemented with 10% fetal bovine serum. The cultured cells were confirmed to be mycoplasma negative by PCR (Mycoplasma detection kit, Sigma, MO). To obtain primary tumor cells, late stage tumors of 16 week old MMTV-PyMT FVB females were harvested and minced using a razor blade, then digested using collagenase A, 2 mg/ml and hyluronidase 100 unit/ml (Worthington, NJ) at 37°C for 1 hour. Cell suspension was passed through 40 um nylon filter (Fisher, PA) before transfer into culture dishes. Non-attached cells and debris were flushed away the second day and the remaining cells were passaged twice with 1 to 5 dilution and cultured for five days before being carefully dissociated to give single cell suspensions and introduced i.v. into host animals.

### Lung Metastasis Assay

8-week-old FVB females or 6-week-old female nude mice were used for all experimental metastasis assays with 5×10^5^ PyMT induced tumor cells and 10^6^ MDA231 derived human tumor cells respectively. If not specified, all animals were sacrificed 2 weeks after I.V. injection of PyMT cells or 4 weeks for human tumor cells for optimal metastatic burden. For paraffin sections, before removal, lungs were injected with 1.2 ml of 10% neutral buffered formalin by tracheal cannulation in order to fix the inner airspaces and inflate the lung lobes. Lungs were then excised and fixed in formalin overnight. A precise stereological method [68] with slight modification was used for lung metastasis quantification. Briefly, paraffin-embedded lungs were systematically sectioned through the entire lung with one 5 um section taken in every 0.5 mm lung thickness. All the sections were stained with H&E and images were taken using a Zeiss SV11 microscope with a Retiga 1300 digital camera and analyzed using ImageJ software. Mac3 (Santa Cruz Biotech., CA) and F4/80 (CALTAG Laboratories) were used for immunohistochemistry staining.

For realtime PCR based quantification of metastatic cells, lungs were harvested at times specified after tumor cell i.v. injection (zero time point was taken at 5 minutes post i.v.), minced, then digested thoroughly with proteinase K. DNA were extracted using DNAeasy Kit (Qiagen). Based on standards prepared in the same way, absolute cell number was quantified by realtime quantitative PCR (Opticon2, MJ/Bio-rad) with specific primers against PyMT transgene. Primers sequences were a kind gift from Dr. Mike Ostrowski (Ohio State University, Ohio).

### Liposomal clodronate preparation and administration

Liposomal encapsulation of clodronate (dichloromethylene diphosphonate) was performed as described previously [25] with slight modification. Briefly, a mixture of 8 mg cholesterol (Avanti, Alabaster, AL) and 86 mg egg-phosphatidylcholine (dioleoyl-phosphatidylcholine, Avanti, Alabaster, AL) was dissolved in methanol/chloroform (1∶9 volume ratio) and then the thin-layer lipid membrane was made by rotor-evaporation under nitrogen protection. The clodronate solution was prepared by dissolving 1.2 g dichloromethylene diphosphonic acid (Sigma Chemical Co.) in 5 ml sterile phosphate-buffered saline, then added to the thin-layer lipid membrane and hydrated for 2 hours at 50 rpm in room temperature. The non-encapsulated chemical was removed by dialysis with Slide-A-Lyzer (Pierce, Rockford IL) and 4L of PBS (refreshed twice in 24 h). The final concentration of clodronate in the liposome formulation was adjusted to 6 mg/ml. A single dose of liposomal clodronate of 0.1 ml/10 g body weight was administered via i.v. or intra-trachea injection at the times specified.

### FACS analysis

For FACS analysis, lungs were perfused briefly with cold PBS before harvest and then minced on ice. Cells were blocked using anti-mouse CD16/CD32 antibody (eBiosciences, CA) before antibody staining. Antibody used are: CD45 (30-F11), CD11b (M1/70), CD11c (HL3), Gr1 (RB6-8C5), CXCR4 (2B11) (BD Pharmingen); Tie2 (TEK4) (eBioscience); CX3CR1 (Torrey Pines Biolabs, TX), VEGFR1 (141522), CCR2 (48607) (R&D systems), and F4/80 (Cl:A3-1) (AbD Serotec). FACS analysis was preformed on a LSRII cytometer (BD biosciences) and data were analyzed using Flowjo software (TreeStar, OR)

### Ex vivo whole lung imaging

A well established intact lung microscopy technique [42], [69] was applied to observe tumor cells, macrophages, and blood vessels in the mouse lungs. CFP-expressing Met-1 cells prepared by retrovirus infection of a CMV promoter-CFP vector were injected i.v. into the tail vein of each mouse. At the time indicated, mice were anesthetized and injected with 10 ug Alexa Fluor® 647 conjugated anti-mouse CD31 antibody (BioLegend, CA). Five minutes later, the mouse was put under artificial ventilation through trachea cannulation. The lung was cleared of blood by gravity perfusion through the pulmonary artery with artificial medium [Krebs-Ringer bicarbonate buffer with 5% dextran and 10 mmol/L glucose (pH 7.4)]. The heart–lung preparation was dissected en bloc and placed in a specially designed plexiglass chamber with a port to the artificial cannula. The lung rested on a plexiglass window at the bottom of the chamber with the posterior surface of the lung touching the plexiglass. The lung was ventilated throughout the experiment with 5% CO_2_ in medical air and perfused by gravity perfusion except during imaging. Three to five animals were imaged for each time point and 10 to 20 unrelated fields were imaged for each animal.

Images were collected with a Leica TCS SP2 AOBS confocal microscope (Mannheim, Germany) with 60×oil immersion optics. Laser lines at 458 nm, 488 nm and 633 nm for excitation of CFP, GFP and AF647 were provided by an Ar laser and a HeNe laser. Detection ranges were set to eliminate crosstalk between fluorophores. Three-dimensional reconstruction was performed using Volocity™ (Improvision Inc., MA) and self-developed macros in ImageJ [70] were used for quantification.

### Statistical analysis

Statistical analysis methods employed were repeated measures ANOVA for primary PyMT tumor cells; standard two-tailed Student's t test for two data sets and ANOVA followed by Bonferroni/Dunn post-hoc tests for multiple data sets using Prism (GraphPad Inc.). p values below 0.05 (*), <0.01 (**) and <0.001 (***) were deemed as significant and highly significant, respectively.

## Supporting Information

Figure S1 Increased apoptosis of metastasizing tumor cells in CSF-1 deficient hosts. Apoptosis (Tunel) and proliferation (Ki67) of metastasizing Met-1 tumor cells in lungs of mice with different genotypes as shown. Apoptosis index (A) and proliferation index (C) are percentage of positive cells in total tumor cells. Data are shown as mean + SEM. nm, ** P<0.01 and *** P<0.001.
(5.33 MB TIF)

Figure S2 In vivo macrophage depletion using in-house-made liposome -encapsulated Clodronate. Representative micrograph of F4/80 immunostaining of liver sections from mice treated with liposome containing PBS (L-PBS, left) and Clodronate (L-Clodronate, right) as described in the materials and methods indicates a dramatic macrophage depletion in the L-Clodronate treated mice. Bar equals 20 um.
(5.25 MB TIF)

Figure S3 Pulmonary macrophage populations are relatively normal in Csf-1op/op mice. (A, C) Representative micrograph of Mac3 stained lung m sections of lungs from mice with resident macrophages in sagittal 5 genotypes shown; (B, D) Representative Giemsa staining of cytospins of alveolar macrophages obtained by lavage in the same mouse genotypes as in A, C, bar = 20um; (E) Graph showing flow cytometric data of percentage of EGFP+ macrophages in total CD45 + cells in lungs of mice of different genotypes designated as in Fig. 1 Data are shown as mean + SEM. n = 3, * p<0.05; (F) Graph showing cytometric data of the percentage of CD11c + CD11b- cells in total Csf-1R-EGFP+ cells in lungs of Tg (Csf1r-eGFP) Hume transgenic mice of different genotypes designated as in Fig. 1. Data are shown as mean + SEM. n = 3.
(11.87 MB TIF)

Figure S4 Lymphocyte and granulocyte populations are not significantly altered in lungs bearing experimental metastasis of Met-1 cells. Bar graphs showing quantitative measurements of flow cytometric data comparing cells with different surface markers as shown from normal lungs and lungs bearing experimentally induced metastasis of Met-1 cells. There were no significant differences between groups. Data are shown as mean + SEM. n = 3.
(4.08 MB TIF)

Figure S5 Lung resident macrophages and metastasis recruited macrophages have similar Mac3 expression. Representative flow histograms of normal lung macrophages (F4/80+, blue) versus recruited macrophage population (F4/80+CD11b+Gr1-, red) from lungs bearing Met-1 cell metastases stained with anti-Mac3 antibodies. X axis indicates the fluorescent intensity, Y axis indicates the percentage of maximum cell number. (n = 3).
(6.88 MB TIF)

Figure S6 CD11b+ macrophages are specifically depleted in CD11b-DTR mosaic mice. Lymphocyte populations in blood of bone marrow chimeras carrying the Diptheria toxin receptor transgene are not affected by DT treatment compared to Glu52-DT treatment while CD11b+ macrophages are specifically depleted by the former treatment. Bar graph showing quantative measurements of flow cytometric data comparing blood cells from CD11b-DTR bone marrow mosaic mice treated with DT or Glu52-DT. Data are shown as mean+ SEM. n = 33, ** P<0.01
(5.05 MB TIF)

Figure S7 CD11b+ Gr1+ cells are not depleted by diphtheria toxin (DT) treatment in vivo. (A) Representative flow diagram of CD45+ cells in lungs of mice in which significant lung metastasis has been established by experimentally introduced Met-1 cells into bone marrow chimeras carrying the Diptheria toxin transgene (Mosaic) or wild type (WT) bone marrow as shown before DT or Glu52-DT treatment. X axis, CD11b expression; Y axis, Gr1 expression.(B) Graph showing flow cytometric data of the percentage of CD11b+ Gr1+ cells in the CD45+ population in lungs of mice in which significant lung metastasis has been established.
(9.79 MB TIF)

Movie S1 Tumor cell attaching to the blood vessel in lung at 5 minutes after tail vein inoculation.
(0.90 MB AVI)

Movie S2 Extravasating tumor cell interacting with macrophages at 24 hours after tail vein inoculation.
(0.81 MB AVI)

Movie S3 Extravasated tumor cell interacting with macrophages outside blood vessel at 48 hours after tail vein inoculation.
(0.79 MB AVI)

Movie S4 Tumor cell cluster interacting with macrophages outside blood vessel at 72 hours after tail vein inoculation.
(0.94 MB AVI)

Movie S5 Tumor cell fail to extravasate with liposome Clodronate treatment depleting interacting macrophage at 24 hours after tail vein inoculation.
(0.61 MB AVI)
