# Supplementary figures and images for: A Distinct Macrophage Population Mediates Metastatic Breast Cancer Cell Extravasation, Establishment and Growth
Source: PLoS One. 2009 Aug 10;4(8):e6562. doi: 10.1371/journal.pone.0006562 (PMC2721818; doi:10.1371/journal.pone.0006562)

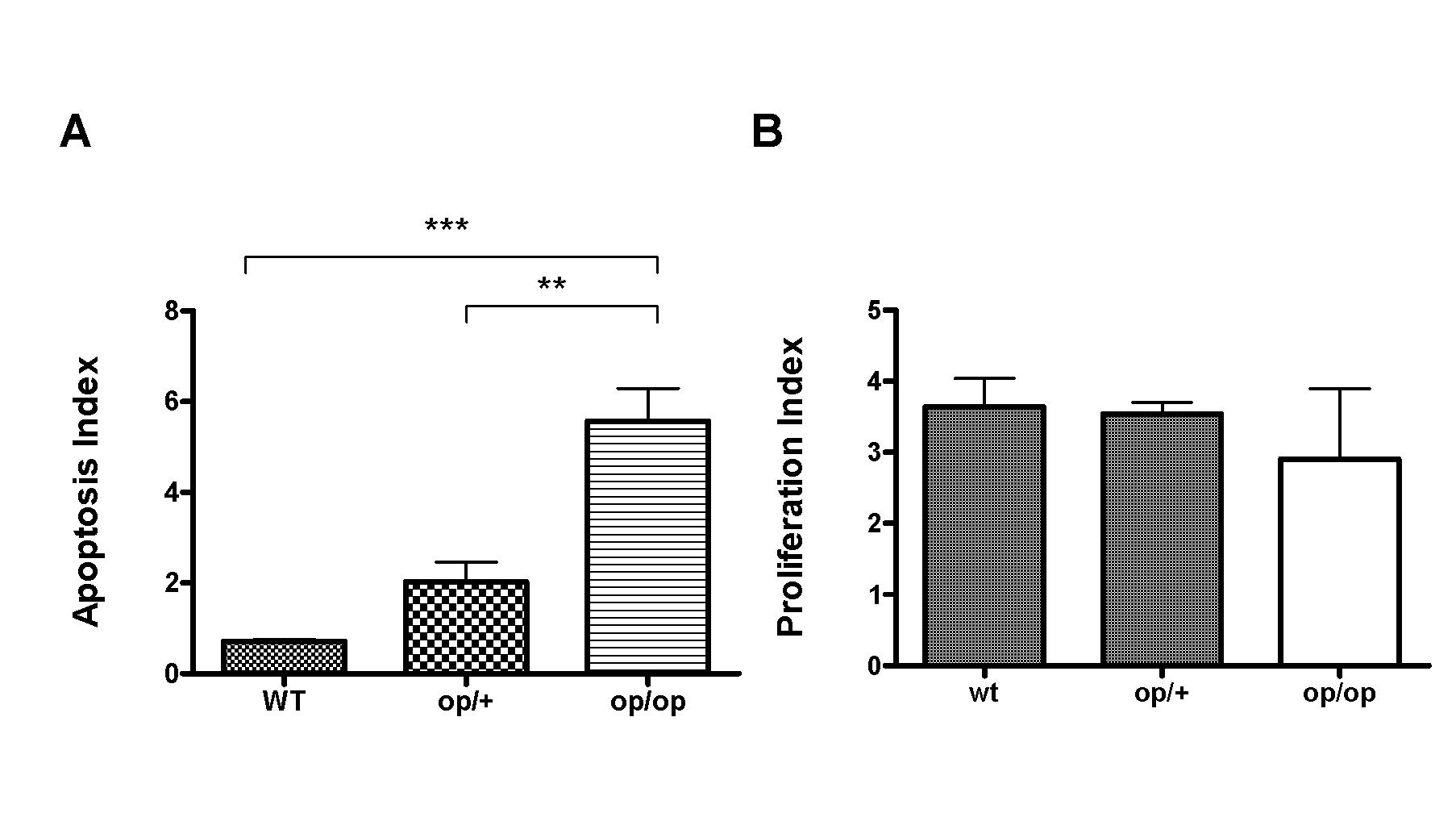

Supplement: Figure S1 — Increased apoptosis of metastasizing tumor cells in CSF-1 deficient hosts. Apoptosis (Tunel) and proliferation (Ki67) of metastasizing Met-1 tumor cells in lungs of mice with different genotypes as shown. Apoptosis index (A) and proliferation index (C) are percentage of positive cells in total tumor cells. Data are shown as mean + SEM. nm, ** P<0.01 and *** P<0.001. (5.33 MB TIF) [file pone.0006562.s001.tif]

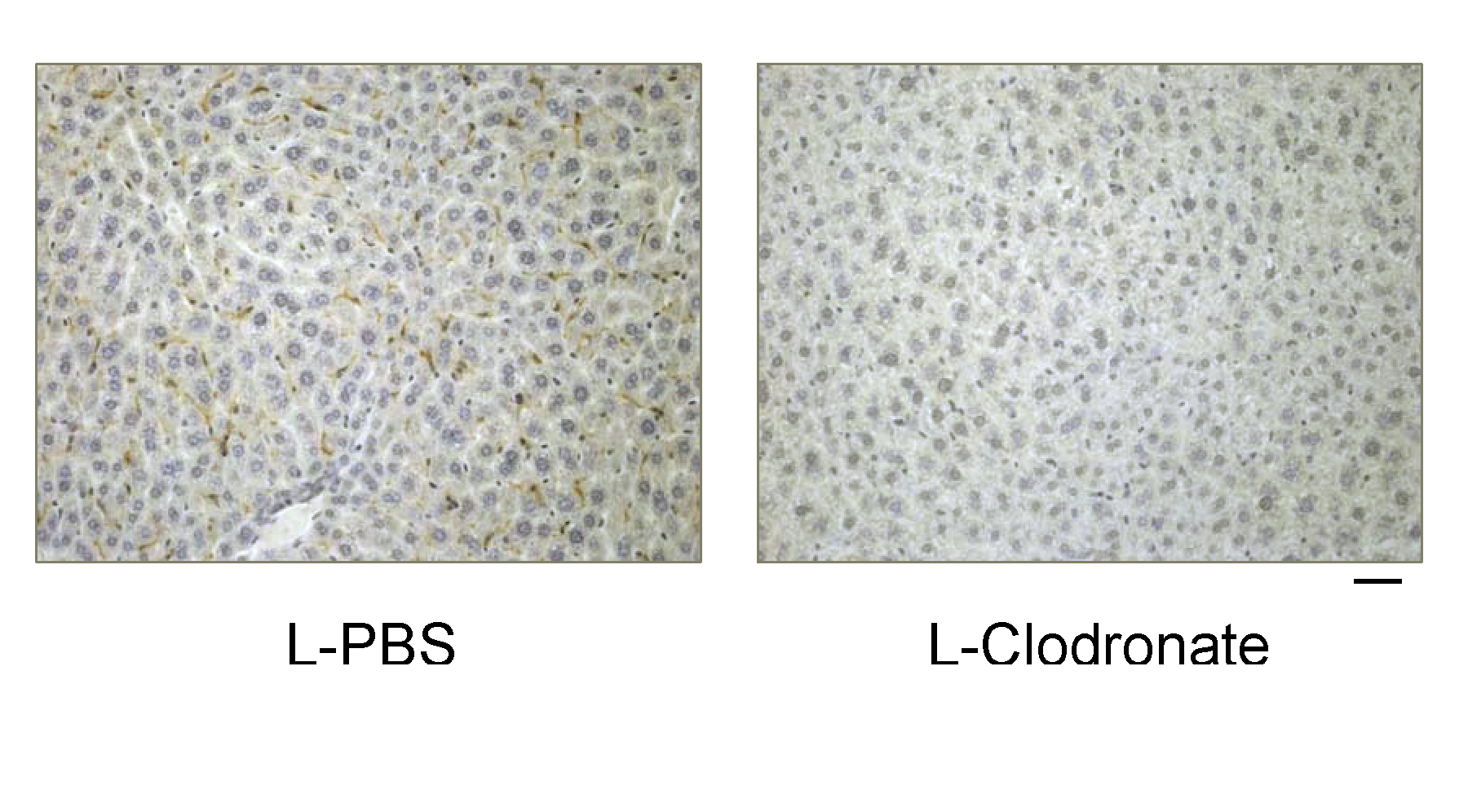

Supplement: Figure S2 — In vivo macrophage depletion using in-house-made liposome -encapsulated Clodronate. Representative micrograph of F4/80 immunostaining of liver sections from mice treated with liposome containing PBS (L-PBS, left) and Clodronate (L-Clodronate, right) as described in the materials and methods indicates a dramatic macrophage depletion in the L-Clodronate treated mice. Bar equals 20 um. (5.25 MB TIF) [file pone.0006562.s002.tif]

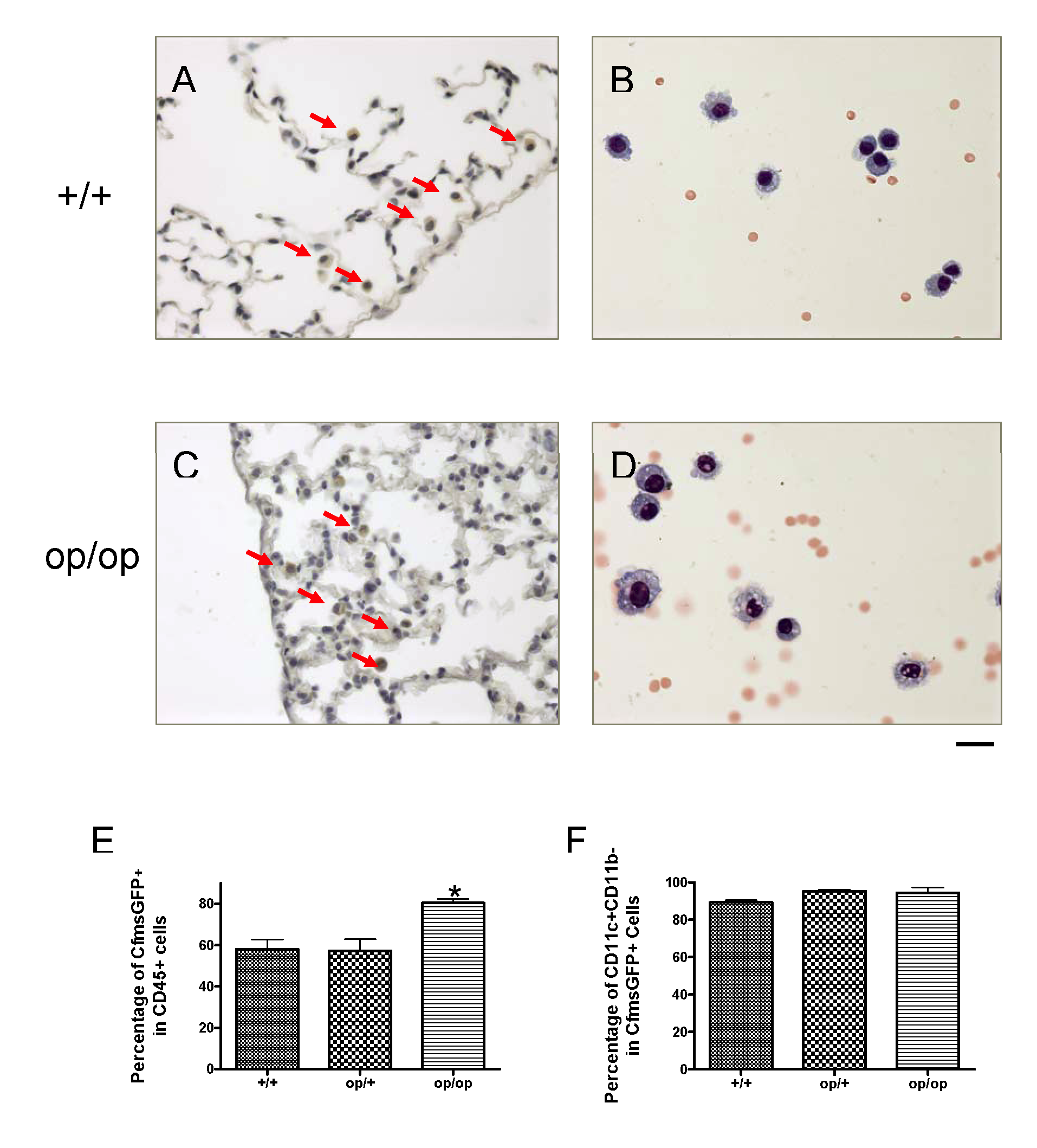

Supplement: Figure S3 — Pulmonary macrophage populations are relatively normal in Csf-1op/op mice. (A, C) Representative micrograph of Mac3 stained lung m sections of lungs from mice with resident macrophages in sagittal 5 genotypes shown; (B, D) Representative Giemsa staining of cytospins of alveolar macrophages obtained by lavage in the same mouse genotypes as in A, C, bar = 20um; (E) Graph showing flow cytometric data of percentage of EGFP+ macrophages in total CD45 + cells in lungs of mice of different genotypes designated as in Fig. 1 Data are shown as mean + SEM. n = 3, * p<0.05; (F) Graph showing cytometric data of the percentage of CD11c + CD11b- cells in total Csf-1R-EGFP+ cells in lungs of Tg (Csf1r-eGFP) Hume transgenic mice of different genotypes designated as in Fig. 1. Data are shown as mean + SEM. n = 3. (11.87 MB TIF) [file pone.0006562.s003.tif]

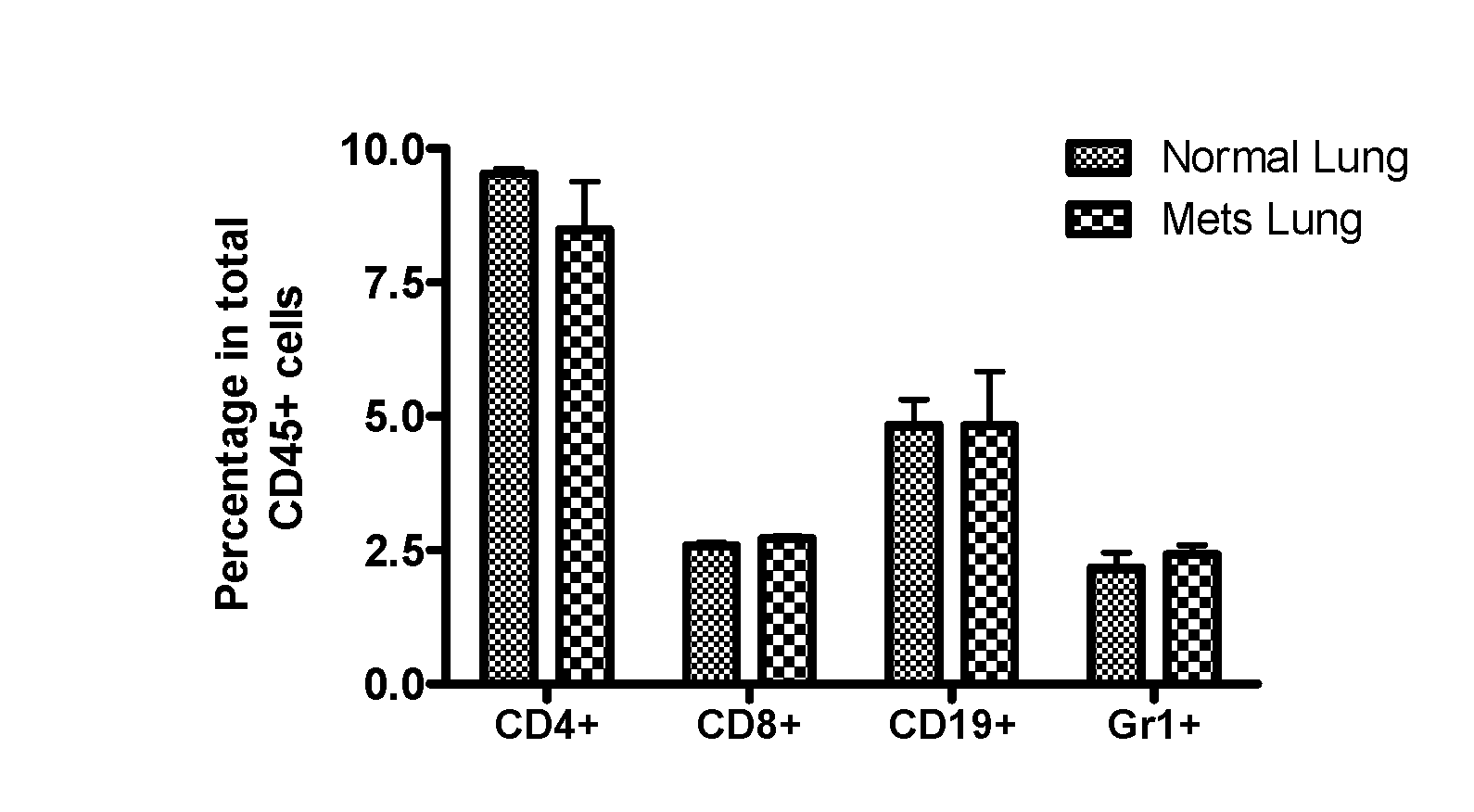

Supplement: Figure S4 — Lymphocyte and granulocyte populations are not significantly altered in lungs bearing experimental metastasis of Met-1 cells. Bar graphs showing quantitative measurements of flow cytometric data comparing cells with different surface markers as shown from normal lungs and lungs bearing experimentally induced metastasis of Met-1 cells. There were no significant differences between groups. Data are shown as mean + SEM. n = 3. (4.08 MB TIF) [file pone.0006562.s004.tif]

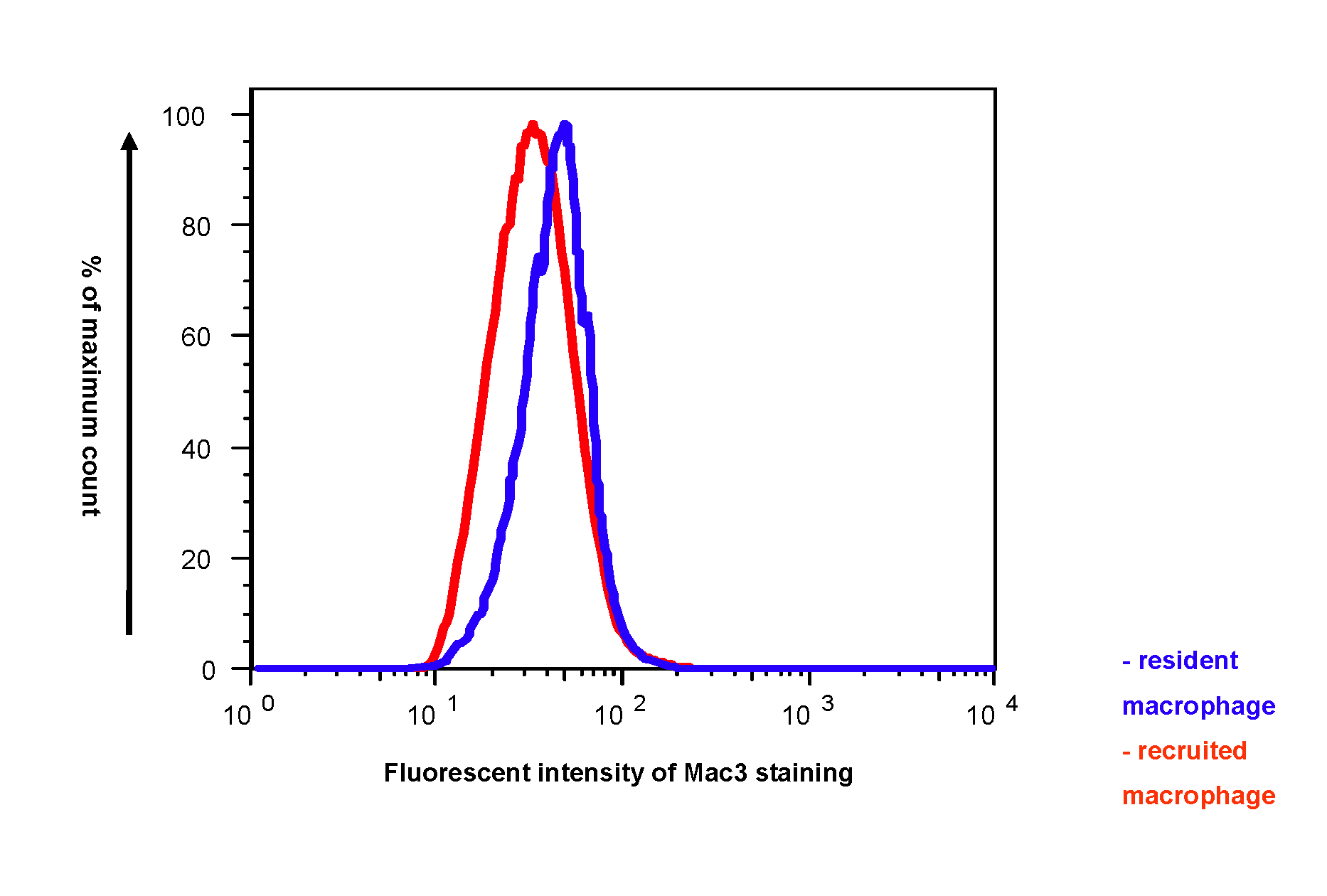

Supplement: Figure S5 — Lung resident macrophages and metastasis recruited macrophages have similar Mac3 expression. Representative flow histograms of normal lung macrophages (F4/80+, blue) versus recruited macrophage population (F4/80+CD11b+Gr1-, red) from lungs bearing Met-1 cell metastases stained with anti-Mac3 antibodies. X axis indicates the fluorescent intensity, Y axis indicates the percentage of maximum cell number. (n = 3). (6.88 MB TIF) [file pone.0006562.s005.tif]

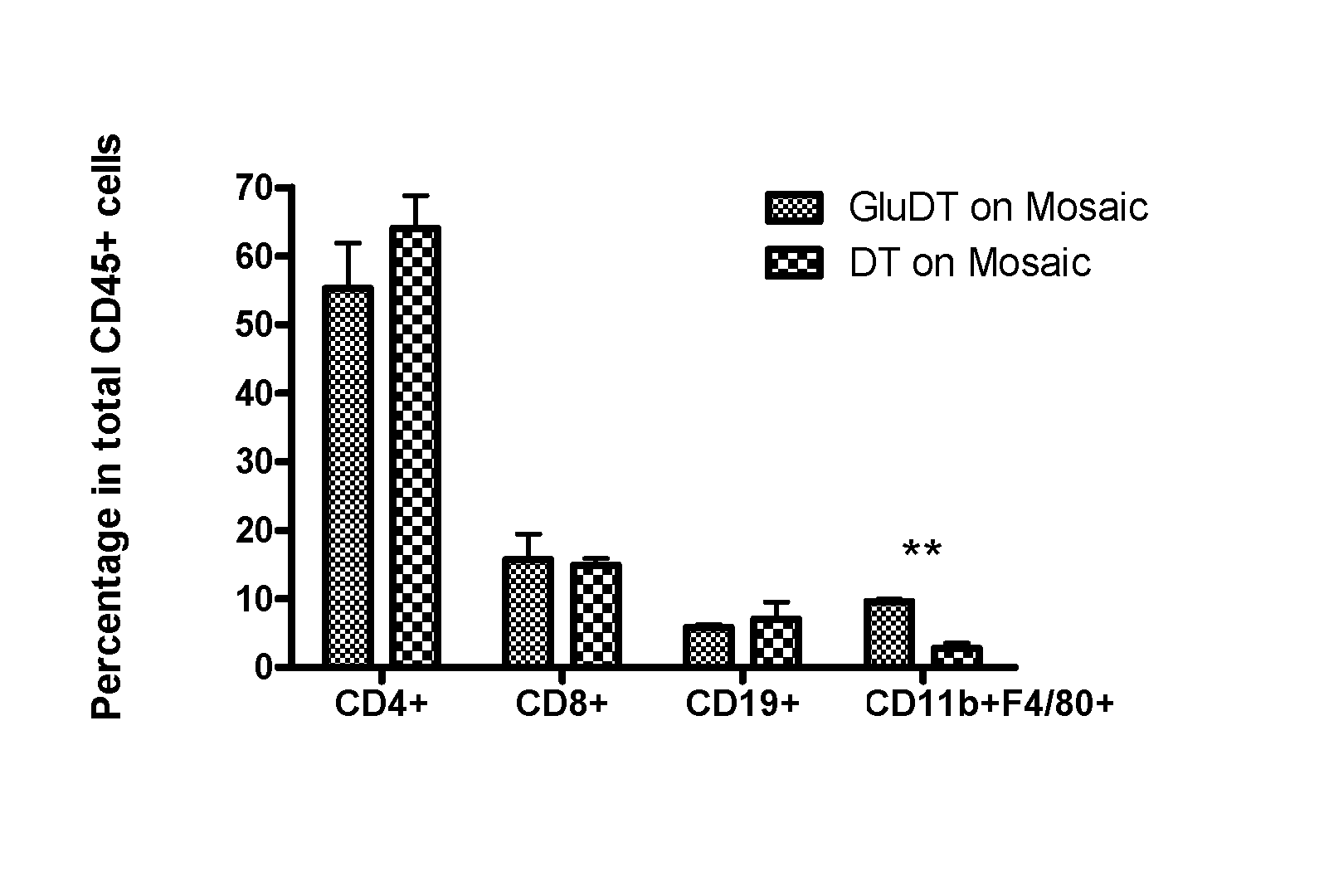

Supplement: Figure S6 — CD11b+ macrophages are specifically depleted in CD11b-DTR mosaic mice. Lymphocyte populations in blood of bone marrow chimeras carrying the Diptheria toxin receptor transgene are not affected by DT treatment compared to Glu52-DT treatment while CD11b+ macrophages are specifically depleted by the former treatment. Bar graph showing quantative measurements of flow cytometric data comparing blood cells from CD11b-DTR bone marrow mosaic mice treated with DT or Glu52-DT. Data are shown as mean+ SEM. n = 33, ** P<0.01 (5.05 MB TIF) [file pone.0006562.s006.tif]

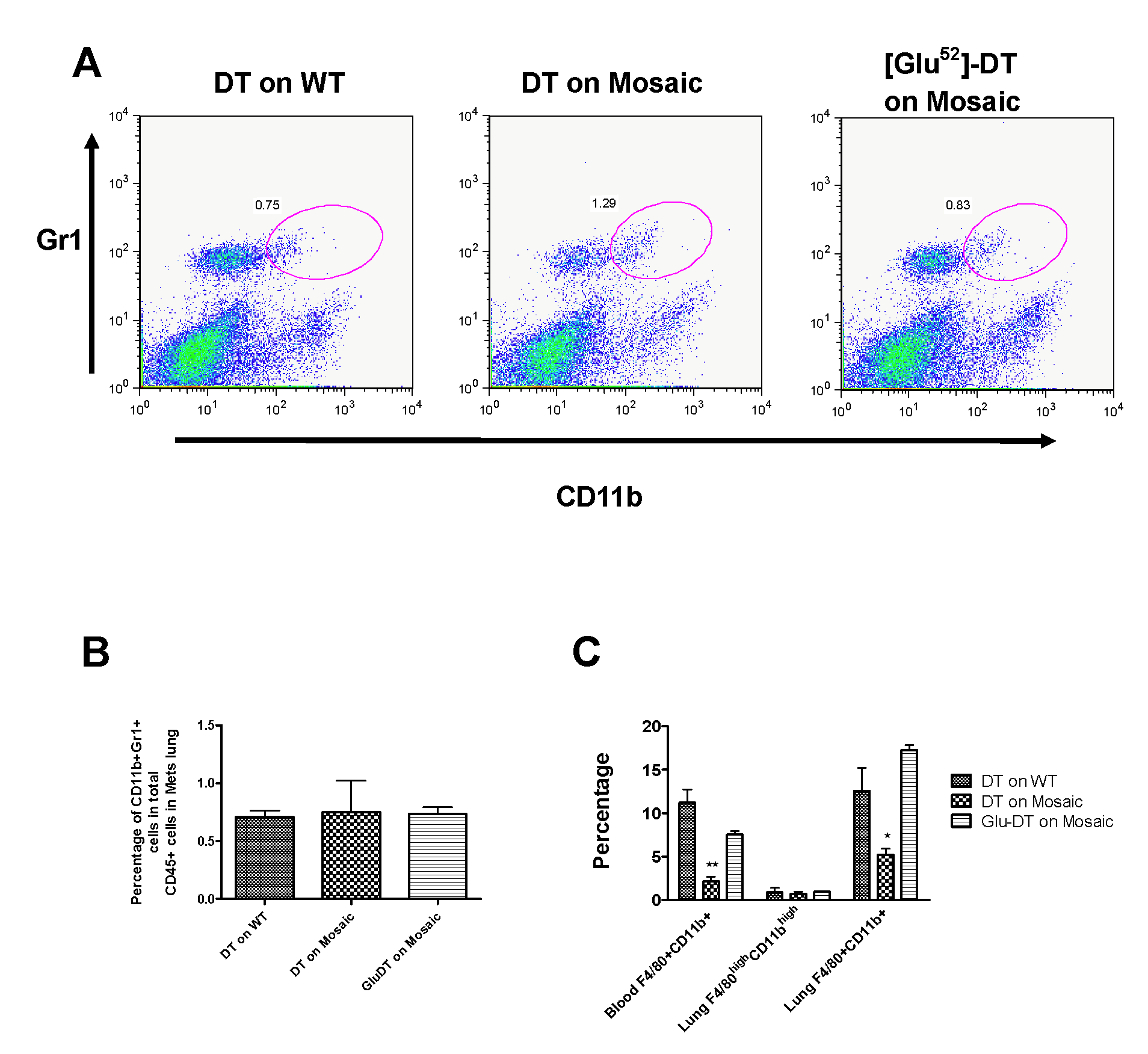

Supplement: Figure S7 — CD11b+ Gr1+ cells are not depleted by diphtheria toxin (DT) treatment in vivo. (A) Representative flow diagram of CD45+ cells in lungs of mice in which significant lung metastasis has been established by experimentally introduced Met-1 cells into bone marrow chimeras carrying the Diptheria toxin transgene (Mosaic) or wild type (WT) bone marrow as shown before DT or Glu52-DT treatment. X axis, CD11b expression; Y axis, Gr1 expression.(B) Graph showing flow cytometric data of the percentage of CD11b+ Gr1+ cells in the CD45+ population in lungs of mice in which significant lung metastasis has been established. (9.79 MB TIF) [file pone.0006562.s007.tif]
